# Systematic Review of Risk Factors Assessed in Predictive Scoring Tools for Drug-Related Problems in Inpatients

**DOI:** 10.3390/jcm11175185

**Published:** 2022-09-01

**Authors:** Lea Jung-Poppe, Hagen Fabian Nicolaus, Anna Roggenhofer, Anna Altenbuchner, Harald Dormann, Barbara Pfistermeister, Renke Maas

**Affiliations:** 1 Institute of Experimental and Clinical Pharmacology and Toxicology, Friedrich-Alexander-Universität Erlangen-Nürnberg, 91054 Erlangen, Germany; 2 University Hospital Erlangen, 91054 Erlangen, Germany; 3 Central Emergency Department, Fürth Hospital, 90766 Fürth, Germany; 4 Hospital Pharmacy, Fürth Hospital, 90766 Fürth, Germany

**Keywords:** risk factors, drug-related problems, adverse drug events, adverse drug reactions, medication errors, predictive scoring tool, clinical pharmacology, clinical pharmacy, risk assessment, risk score

## Abstract

Drug-related problems (DRP, defined as adverse drug events/reactions and medication errors) are a common threat for patient safety. With the aim to aid improved allocation of specialist resources and to improve detection and prevention of DRP, numerous predictive scoring tools have been proposed. The external validation and evidence for the transferability of these tools still faces limitations. However, the proposed scoring tools include partly overlapping sets of similar factors, which may allow a new approach to estimate the external usability and validity of individual risk factors. Therefore, we conducted this systematic review and analysis. We identified 14 key studies that assessed 844 candidate risk factors for inclusion into predictive scoring tools. After consolidation to account for overlapping terminology and variable definitions, we assessed each risk factor in the number of studies it was assessed, and, if it was found to be a significant predictor of DRP, whether it was included in a final scoring tool. The latter included intake of ≥ 8 drugs, drugs of the Anatomical Therapeutic Chemical (ATC) class N, ≥1 comorbidity, an estimated glomerular filtration rate (eGFR) <30 mL/min and age ≥60 years. The methodological approach and the individual risk factors presented in this review may provide a new starting point for improved risk assessment.

## 1. Introduction

Drug-related problems (DRP) such as adverse drug events/reactions (ADE/ADR) and medication errors (ME) have been recognized as a common and serious threat for patient safety. They are an important cause of morbidity and mortality, can provoke hospital admissions as well as prolong the length of stay, and, thereby, increase costs in the health care system [1,2,3,4,5]. In clinical routines, a significant proportion of DRP is detected (too) late or even remains undetected [6], leading to further unnecessary suffering and costs. In principle, an independent medication review by a specialist in medication safety (i.e., a (clinical) pharmacist or clinical pharmacologist) may help to detect, resolve, and, at best, prevent DRP. However, in many health care settings, these resources are limited. This results in a great interest to devise simple scoring systems to prescreen patients by non-specialists or with the help of software tools in order to identify patients at the highest risk for DRP and thus most likely to benefit from the allocation of scarce specialist attention.

In recent years, several of these predictive scoring tools have been developed. Plenty of literature reviews evaluating the overall performance and quality of these predictive scoring tools have already been published [7,8,9,10,11,12,13]. These reviews frequently came to the conclusion that—limited external validation aside—the performance of the existing predictive scoring tools for drug-related problems is at best moderate and that the scores are often not easily applicable to other settings. In addition, the proposed scores not only show significant variation, but also some overlap regarding the set and number of individual factors they consider. Therefore, new approaches are needed to not only identify and validate the overall predictive scoring tools, but also to identify and externally validate the individual risk factors these scoring tools assess.

While the majority of scoring tools may lack extensive external validation of the whole tool, some individual risk factors appear to be components of many risk scores, and as such, individual risk factors may have been validated in a much larger number of studies than the overall score. A comprehensive assessment of publications regarding which risk factors have been investigated and which of these have been included most frequently into a final scoring tool may give an indication of their predictive value as well as their overall applicability to identify patients at risk for DRP. Moreover, numerous potential risk factors have already been assessed for inclusion into risk scores but were found unsuitable. Prior knowledge of these most-likely unsuccessful candidate risk factors could help to save resources in future studies.

Therefore, the primary aim of the present systematic review was to comprehensively screen publications devising and evaluating predictive risk scoring tools in order to identify individual risk factors that have repeatedly been assessed and confirmed (or refuted) as suitable components of scoring tools used to identify patients at risk for DRP. The individual risk factors identified by this approach, as well as the methodological approach itself, are intended to aid and improve the development of future risk scoring tools.

## 2. Materials and Methods

This systematic review was conducted and reported according to the Preferred Reporting Items for Systematic Reviews and Meta-Analyses (PRISMA) [14] and follows the methods described in the protocol, which is available in Supplementary File S1. The protocol was not registered.

### 2.1. Definitions

For simplification, in this review we used the term “predictive scoring tool” when referring to any kind of risk scores and scales, prognostic models, algorithms, and clinical decision rules.

### 2.2. Inclusion and Exclusion Criteria

Studies were included if they were published in the period between 2011 and August 2021 and discussed the development of a general predictive scoring tool for the identification of inpatients at risk for drug-related problems (DRP), comprised of adverse drug events (ADE) and/or adverse drug reactions (ADR) and medication errors (ME) irrespective of the statistical approach. It is a well-known issue in the field of medication safety that a multitude of definitions are in use for DRP, ADE, ADR, and ME (as reviewed elsewhere) [15,16]. Given the general overlap of the definitions used by studies retained for final analysis, we deemed their combined assessment acceptable for the purpose of this analysis but indicate possible limitations in the discussion and list the terms and definitions as provided by the authors of the respective publications in Supplementary File S2.

Among the exclusion criteria were predictive scoring tools applied by patients themselves (e.g., via questionnaire), articles investigating medical errors made by nurses, articles only identifying risk factors for DRP without developing a predictive scoring tool and predictive scoring tools only focusing on a specific demographic patient population (except geriatric patients), patients with specific conditions, and only a single step in the medication process or a specific DRP (e.g., only bleeding due to vitamin k antagonists). Scoring tools using other outcomes than DRP (e.g., inappropriate prescribing, medication discrepancies) were also excluded.

A detailed list containing all inclusion and exclusion criteria is provided in Supplementary File S3.

### 2.3. Information Sources and Search Strategies

A literature search was performed in three different databases: National Library of Medicine PubMed^®^ (last consulted 9 August 2021), Cochrane Library, and Scopus (both last consulted 10 August 2021).

The search strategy in PubMed combined Medical Subject Headings (MESH) terms and free text search with Boolean operators, whereas the search strategy in Scopus and Cochrane Library included free text search and Boolean operators. Please see Supplementary File S4 for the search strategies of each database in detail. A filter for the period of time (January 2011 to August 2021) was used for all three databases. Additionally, we undertook a hand search in Google and a hand search of citations of all studies that met the inclusion and exclusion criteria as well as external validations of the included scoring tools.

### 2.4. Selection Process

Five authors (L.J.-P., H.F.N., A.R., B.P., and R.M.) were involved in the selection process for eligible studies, which was conducted according to the PRSIMA statement [14]. After removal of review articles, duplicates, and articles in other languages than English or German, all remaining records identified from the databases were screened by title. Appropriate records were then screened by abstract for an association with risk factors for DRP. Subsequently, full texts of the reports retrieved for eligibility were evaluated using the prespecified inclusion and exclusion criteria. A full text evaluation of the records identified from citation search and hand search in Google was undertaken accordingly.

### 2.5. Data Collection and Risk Bias Assessment

The first authors (L.J.-P. and H.F.N.) reviewed all studies retrieved for eligibility independently to ensure a valid and reproducible process. The following data items were extracted from the included studies: First author, year of publication, name of the predictive scoring tool, country, study design, study population and setting, sample size, outcome, number of risk factors in the scoring tool, method of identification of risk factors, model development and performance, internal and external validation, and all risk factors considered for inclusion. For unclear and missing information, corresponding authors were contacted directly. In case of uncertainty, a co-author (B.P. or A.R.) was consulted.

### 2.6. Synthesis Methods

**Figure 1:**
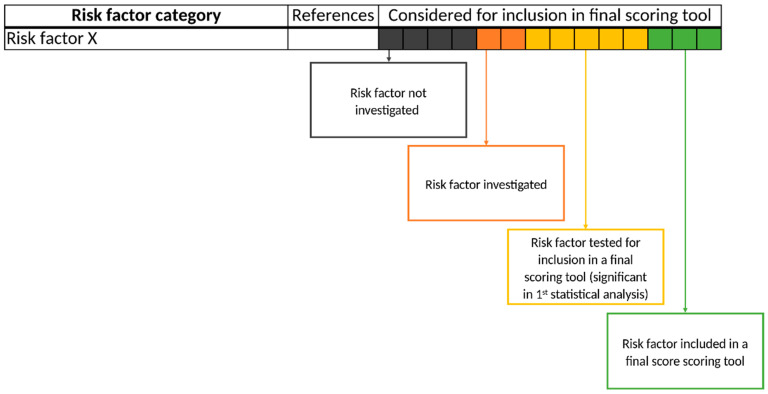
Schematic presentation of risk factors in the form of a color-coded table. Each of the 14 boxes represents one of the included studies. The color of the boxes portrays whether a risk factor was not investigated in a study (gray), was investigated (orange), was significant in a first statistical analysis or based on expert consensus and therefore tested for inclusion in a final scoring tool (yellow), or whether a risk factor was included in a final scoring tool (green). The “citations”-column lists each study that at least investigated the respective risk factor.

All risk factors named in the included publications were listed regardless of inclusion in the final predictive scoring tool. Then, risk factors were classified into six dimensions to obtain a clear arrangement and structure: Drug-related risk factors, diagnosis-related risk factors, laboratory value-related risk factors, vital sign-related risk factors, patient-related risk factors, and medication process/hospital setting-related risk factors. For each identified risk factor, we first evaluated whether the risk factor had been investigated by the authors of the included studies. If investigated, we assessed whether the risk factor was significant in the statistical analysis applied by the authors, and therefore tested for inclusion in the final scoring tool. Finally, we determined the inclusion of the risk factor in the final scoring tool. These findings are portrayed in color-coded tables (Figure 1).

Since risk factors of one dimension were often named inconsistently, were approached in either a more generalized or a more specific manner, and/or different thresholds or definitions were applied, further categorization was needed. To resolve this issue, we summarized isolated risk factors into specific subcategories and more general categories using a bottom-up approach. For example, the isolated risk factors “chronic heart failure (defined by Charlson comorbidity index)” [17], “heart failure” [18], and “congestive cardiac failure“ [19] were summarized in the subcategory, “heart failure”. This subcategory, as well as the subcategories “myocardial infarction”, “ischemic heart disease”, “atrial fibrillation”, “hypotension”, and “hypertension”, form the general category, “cardiovascular diseases”. The assessment of a general category as a risk factor for DRPs was counted if either the authors directly investigated the general category (e.g., “disease of the cardiovascular system” [20]) or if they investigated a subcategory of the general category, thereby fulfilling the criteria of the more general category (e.g., “heart failure” [18]).

Some risk factors included in scoring tools were found to even reduce the risk of occurrence of a DRP [20,21], and others simply did not increase the risk (e.g., “number of drugs ≤ 5” counts as 0 points in the GerontoNet ADR risk score [22]). We assigned these risk factors to their respective category and recorded them as “investigated by the authors” but not as “included in the final score” because we only focused on risk factors that increase the risk of a DRP in this review.

## 3. Results

### 3.1. Study Selection

A total of 2319 studies were identified through the database search. After removal of duplicates, review articles, and publications in languages other than English or German, 2092 records were screened by title and, when appropriate, by abstract. Of these, only 52 were associated with risk factors for DRP. Full text evaluation resulted in 10 studies describing the development of a predictive scoring tool. Another publication [23] was used as a source of risk factors as it describes the selection process of the risk factors for one of the predictive scoring tools [24].

**Figure 2:**
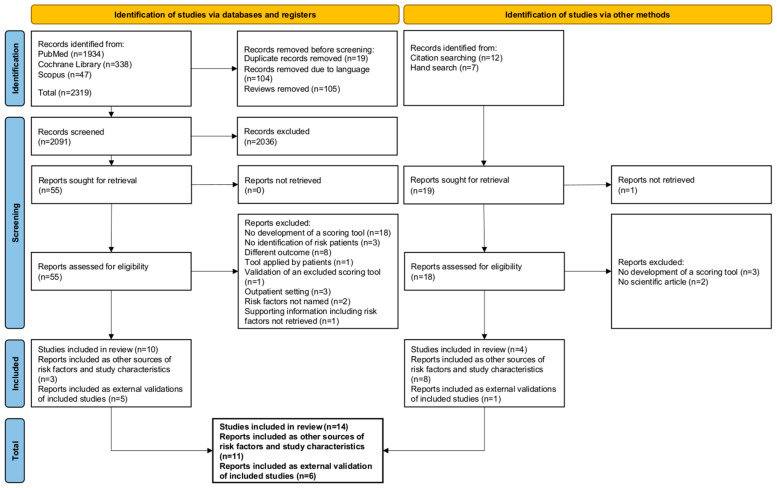
Study selection process displayed as a PRISMA flow diagram.

An additional 14 records were identified through hand search of citations and hand search in Google, of which 4 were included in the systematic review as predictive scoring tools and 2 as additional sources of risk factors (Figure 2).

One predictive scoring tool [22] was found via one of its external validation studies, which was identified from our regular literature search [25]; since it is a frequently cited study and met all other inclusion criteria, we included it in the systematic review even though it was published in 2010.

### 3.2. Study Characteristics

The characteristics of the 14 studies are presented in Table 1. Both prospective and retrospective approaches, as well as a combination of both, were chosen as study designs. The studies were conducted at a multitude of different wards within the hospitals. The most common were internal medicals wards [17,18,19,20,22,24,26,27,28], surgical wards [17,18,27,28,29], geriatric wards [19,20,22,24,30], and emergency departments (ED) [20,31]. Regarding the study population, patients of at least 18 years of age were accepted in most studies. However, some investigators developed predictive scoring tools for use in older patients [18,19,22,30] or used historic populations consisting of older patients [26,32,33] and therefore included only patients of at least 65 years of age. Sakuma et al., on the other hand, even included patients that were at least 15 years of age [27]. Overall, the sample size varied between 303 [26] and 10,800 [28] patients. Furthermore, different methods for internal validation were applied. For instance, some studies used validation via bootstrap samples [20,21,29,30], whereas others set up extra validation sets [17,18,19,22,26,27,28]. Six of the 14 studies were also validated externally [19,22,26,28,31,34]. Outcome measurements, for which the predictive scoring tools were calculated, varied broadly throughout the studies from DRP [20,28] to ME [21,26,34] to ADE [24,27,29,30,31,34] and ADR [17,18,19,22,31] (Supplementary File S2). While a few studies only investigated prespecified risk factors identified via literature search and/or expert consensus [26,34], or only risk factors identified by statistical analysis of the patient characteristics [17,21,27,28,30], many chose a combination of these two methods for risk factor identification and evaluation [18,19,20,22,24,29,31].

In one case, the study design as well as information about the study population and sample size were not given, as it is a practice report [34].

### 3.3. Risk Factors

Added together, we were able to identify a total of 844 risk factors that were investigated for inclusion in a predictive scoring tool by the 14 studies. As detailed in the methods, these factors were summarized into categories that are presented below.

For presentation, isolated risk factors (*n* < 2) that did not match with any of the categorized risk factors or were not related to an existing sub- and/or general category were excluded from the count but can still be seen in Supplementary File S5.

#### 3.3.1. Drug-Related Risk Factors

**Figure 3:**
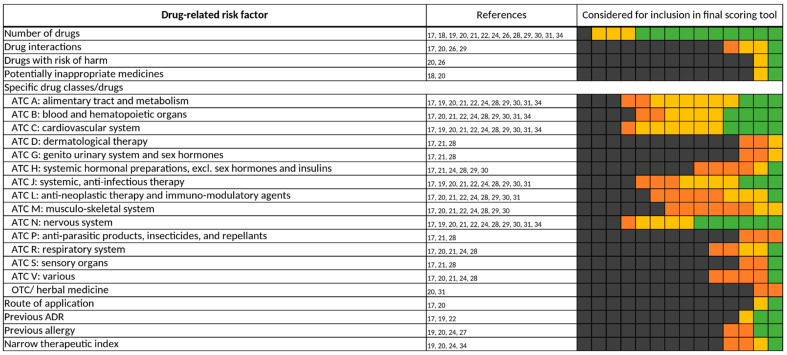
Drug-related risk factors. Specific drugs and drug classes were assigned according to their respective Anatomical Therapeutic Chemical (ATC) Classification System codes. ADR, adverse drug reaction; OTC, over-the-counter. Each of the 14 boxes represents one of the included studies. The color of the boxes portrays whether a risk factor was not investigated in a study (gray), was investigated (orange), was significant in a first statistical analysis or based on expert consensus and therefore tested for inclusion in a final scoring tool (yellow), or whether a risk factor was included in a final scoring tool (green). The “citations”-column lists each study that at least investigated the respective risk factor.

**Figure 4:**
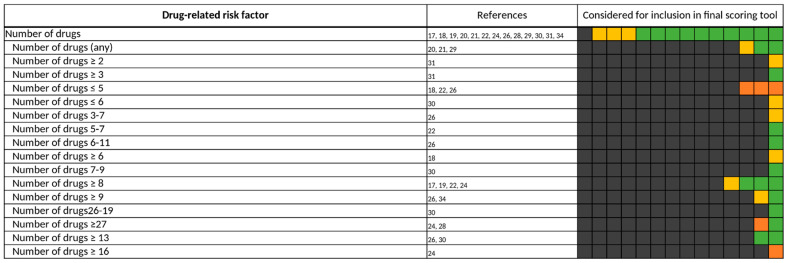
All different thresholds investigated by the authors of the included studies for “number of drugs”. Each of the 14 boxes represents one of the included studies. The color of the boxes portrays whether a risk factor was not investigated in a study (gray), was investigated (orange), was significant in a first statistical analysis or based on expert consensus and therefore tested for inclusion in a final scoring tool (yellow), or whether a risk factor was included in a final scoring tool (green). The “citations”-column lists each study that at least investigated the respective risk factor.

For drug-related risk factors, and even in general, “number of drugs” is one of the most frequently assessed (13/14) [17,18,19,20,21,22,24,26,28,29,30,31,34] and included risk factor (10/13) [19,20,21,22,24,26,28,30,31,34] (Figure 3). Only Sakuma et al. did not investigate “number of drugs” for their predictive scoring tool [27]. In Bos et al. and O’Mahony et al., “number of drugs” was not included in the final scoring tool due to its strong correlation with the risk factors, “age” and “comorbidities”, while Lima et al. only included “number of drugs” depending on the method of administration (≥6 oral drugs and/or ≥1 intravenous drug) [17,18,29]. Though frequently assessed, the threshold for “number of drugs” that was considered in candidate risk factors varied across the studies from 0 [18,22,26,30] to over 16 drugs [24] (Figure 4). 

**Figure 5:**
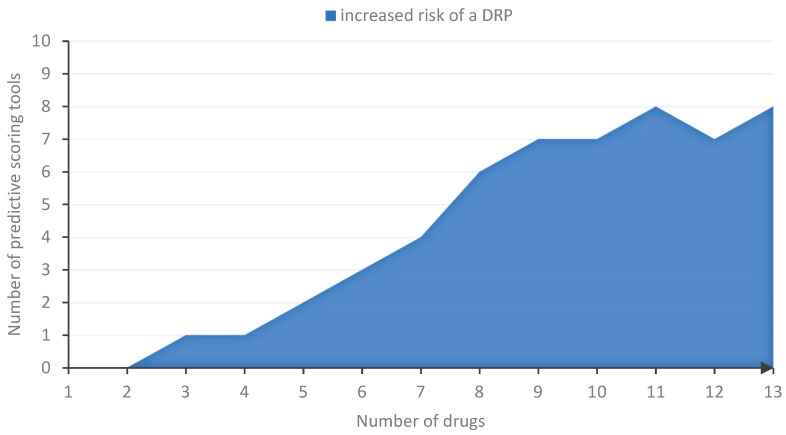
Thresholds associated with an increased risk of DRP based on number of drugs. For every number of drugs, we determined the number of final scoring tools that covered that number by their included threshold for “number of drugs”.

Bos et al., Geeson et al., and Nguyen et al. gave no specific threshold for the number of drugs [20,21,29]. In more than half of the studies that included a defined threshold for number of drugs in their respective scoring tool, eight or more drugs were considered to increase the risk of a DRP (Figure 5). A total number of less than two drugs was never included in a final predictive scoring tool, while a number of less than five drugs was included in two scoring tools [22,26] but was considered to not increase the risk of a DRP.

**Figure 6:**
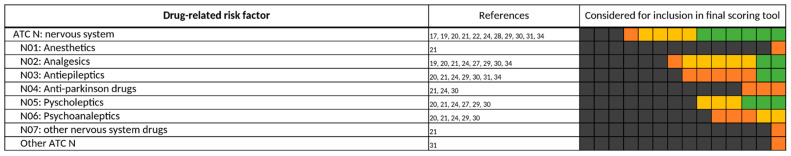
Drug classes of the ATC N (nervous system) class. Each of the 14 boxes represents one of the included studies. The color of the boxes portrays whether a risk factor was not investigated in a study (gray), was investigated (orange), was significant in a first statistical analysis or based on expert consensus and therefore tested for inclusion in a final scoring tool (yellow), or whether a risk factor was included in a final scoring tool (green). The “citations”-column lists each study that at least investigated the respective risk factor.

We (re-)assigned specific drugs and drug classes according to their respective Anatomical Therapeutic Chemical (ATC) Classification System codes. Almost every scoring tool contains at least one specific drug class as a risk factor for DRPs [17,19,20,21,24,27,28,29,30,31,34]. The most frequently investigated ATC classes were ATC A, B, C, J, M, L, N. Within the ATC classes, the specific drug classes examined in the 14 studies were diverse. For ATC A (alimentary tract and metabolism), antidiabetics were assessed in nine studies but were only included in the two final scoring tools, “ART” and “BADRI” [19,34]. For ATC B (blood and hematopoietic organs), mainly anticoagulants were investigated [20,24,27,29,30,34]. Any drugs of the ATC C (cardiovascular system) category were assessed in 11 studies [17,19,20,21,24,27,28,29,30,31,34], but were only included in 4 final scoring tools [24,28,29,34]. Of those included, mainly the whole ATC C drug class was considered to increase the risk of DRPs [28,29,34], and only for a specific drug class (anti-arrhythmics) [24]. Drugs of the ATC N (nervous system) class were included in more than half of the studies examining this risk factor [20,21,24,29,34]. Here, opioids, anti-epileptics, and antipsychotics were the most frequently assessed drug classes of ATC N (Figure 6). On the contrary, drugs of the ATC L (anti-neoplastic therapy and immuno-modulatory agents) [17,20,21,24,27,28,29,30,31,34] and ATC M (musculo-skeletal system) class [17,21,24,27,28,29,30,31] were often assessed as a risk factor for the occurrence of a DRP but were rarely included in a final scoring tool [24].

#### 3.3.2. Diagnosis-Related Risk Factors

**Figure 7:**
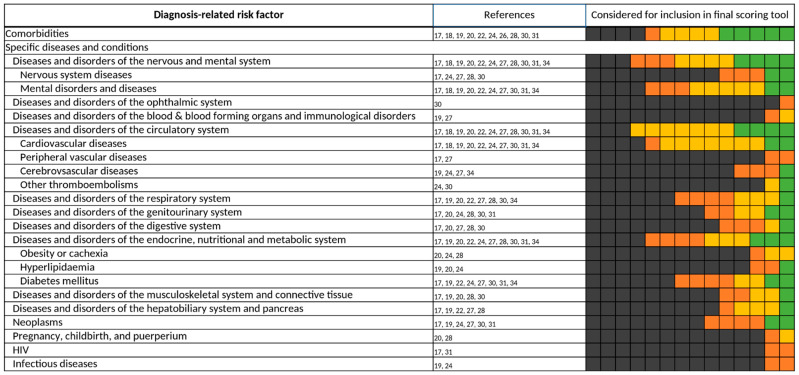
Diagnosis-related risk factors. Specific diseases and conditions were assigned according to their respective Major Diagnostic Categories (MDC). Each of the 14 boxes represents one of the included studies. The color of the boxes portrays whether a risk factor was not investigated in a study (gray), was investigated (orange), was significant in a first statistical analysis or based on expert consensus and therefore tested for inclusion in a final scoring tool (yellow), or whether a risk factor was included in a final scoring tool (green). The “citations”-column lists each study that at least investigated the respective risk factor.

Comorbidities were frequently investigated (10/14) [17,18,19,20,22,24,26,28,30,31] and included (5/10) [18,20,22,28,31] in a final scoring tool (Figure 7). Yet, assessment of comorbidities differed from each study—some used the number of comorbidities [18,19,20,22,24,26,30,31], and others used the Charlson Comorbidity Index (CCI) [17,20,26,28], Diagnosis-related Groups (DRG) weight [20,28], or the modified cumulative illness rating scale (mCIRS) [18].

Likewise, different definitions of diagnoses were set throughout the studies, classifying diseases, for example, by the International Statistical Classification of Diseases and Related Health Problems (ICD), Major Diagnostic Categories (MDC), chronic care management (CCM) program, organ-based approaches, or symptoms. For standardization, we reassigned each considered diagnosis to their respective MDC class.

Most scoring tools contain at least one specific disease [17,19,20,22,24,27,28,31,34]. Diseases of the nervous and mental system [17,18,19,20,22,24,27,28,30,31,34], diseases of the circulatory system [17,18,19,20,22,24,27,28,30,31,34], and diseases of the endocrine system [17,19,20,22,24,27,28,30,31,34] were the most frequently assessed disease classes. Among the diseases of the circulatory system, cardiovascular diagnoses [17,18,19,20,22,24,27,30,31,34], and especially congestive heart failure [17,18,19,22,24,27,31,34], were commonly investigated, whereas diabetes mellitus [17,19,22,24,27,30,31,34] was the most common among the diseases of the endocrine system.

#### 3.3.3. Laboratory Value-Related Risk Factors

**Figure 8:**
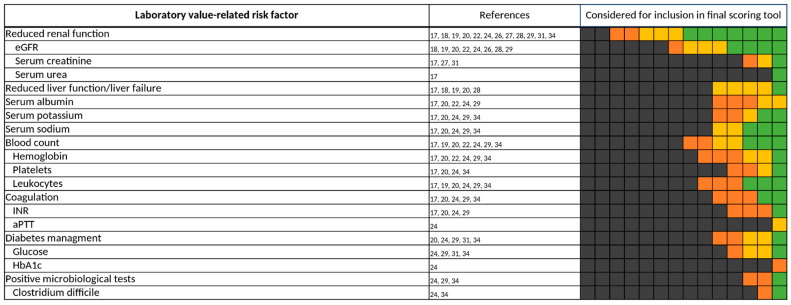
Laboratory value-related risk factors. Each of the 14 boxes represents one of the included studies. The color of the boxes portrays whether a risk factor was not investigated in a study (gray), was investigated (orange), was significant in a first statistical analysis or based on expert consensus and therefore tested for inclusion in a final scoring tool (yellow), or whether a risk factor was included in a final scoring tool (green). The “citations”-column lists each study that at least investigated the respective risk factor.

The renal function—mainly displayed as eGFR—was most frequently assessed [17,18,19,20,22,24,26,27,28,29,31,34] and included [17,18,20,22,26,31,34] in a final predictive scoring tool among the laboratory-value-related risk factors shown in Figure 8. However, thresholds and formulas for the calculation of the eGFR varied widely throughout the studies. Liver function, on the other hand, was rarely assessed [17,18,19,20,28] and only included once [18] in a final scoring tool. In analogy to renal function, the assessment of liver function was inhomogeneous in the studies and authors often referred to different thresholds for elevated liver enzymes and bilirubin [17,18,20] or did not give a specific definition for reduced liver function [19,28]. Other laboratory value-related risk factors investigated in the 14 studies contained serum electrolytes [17,20,24,29,34], albumin [17,20,22,24,29], blood count [17,19,20,22,24,29,34], coagulation [17,20,24,29,34], diabetes-related laboratory values [20,24,29,31,34], and microbiological tests [24,29,34].

#### 3.3.4. Vital Sign-Related Risk Factors

**Figure 9:**
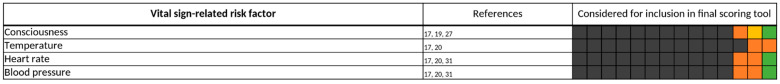
Vital sign-related risk factors. Each of the 14 boxes represents one of the included studies. The color of the boxes portrays whether a risk factor was not investigated in a study (gray), was investigated (orange), was significant in a first statistical analysis or based on expert consensus and therefore tested for inclusion in a final scoring tool (yellow), or whether a risk factor was included in a final scoring tool (green). The “citations”-column lists each study that at least investigated the respective risk factor.

Vital sign-related risk factors (Figure 9) were rarely assessed and were only included in the final scoring tool of Lima et al. and Sakuma et al. [17,27]. The investigated risk factors contained risk factors of the categories, consciousness [17,19,27], temperature [17,20], heart rate [17,20,31], and blood pressure [17,20,31].

#### 3.3.5. Patient-Related Risk Factors

**Figure 10:**
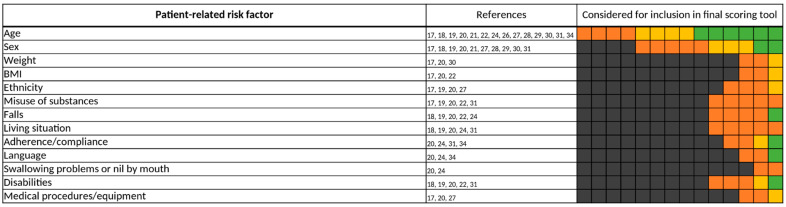
Patient-related risk factors. Each of the 14 boxes represents one of the included studies. The color of the boxes portrays whether a risk factor was not investigated in a study (gray), was investigated (orange), was significant in a first statistical analysis or based on expert consensus and therefore tested for inclusion in a final scoring tool (yellow), or whether a risk factor was included in a final scoring tool (green). The “citations”-column lists each study that at least investigated the respective risk factor.

**Figure 11:**
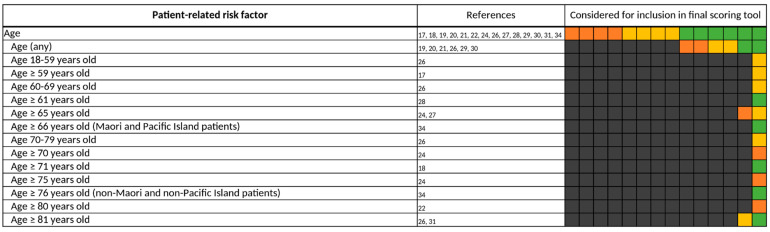
All different thresholds investigated by the authors of the included studies for “age”. Each of the 14 boxes represents one of the included studies. The color of the boxes portrays whether a risk factor was not investigated in a study (gray), was investigated (orange), was significant in a first statistical analysis or based on expert consensus and therefore tested for inclusion in a final scoring tool (yellow), or whether a risk factor was included in a final scoring tool (green). The “citations”-column lists each study that at least investigated the respective risk factor.

Of all risk factors, “age” was the most frequently investigated, being considered for inclusion in a predictive scoring tool by all 14 studies [17,18,19,20,21,22,24,26,27,28,29,30,31,34] (Figure 10). However, thresholds varied widely (Figure 11) and were often predetermined by the setting of the study, e.g., by only including patients admitted to geriatric wards [19,20,22,24,30]. Bos et al., Geeson et al., Nguyen et al., Tangiisuran et al., and Trivalle et al. did not specify a threshold for “age” but instead applied “age” as a continuous variable [19,20,21,29,30]. If a threshold was defined, most authors considered elderly patients of at least 60 years of age at risk for the occurrence of a DRP. Patients’ sex was also frequently assessed, especially considering the female sex as a risk factor for DRPs in 7 of the 14 studies [17,18,19,21,28,30,31], but it was only included twice [17,18] in a final scoring tool.

#### 3.3.6. Medication Process/Setting-Related Risk Factors

**Figure 12:**
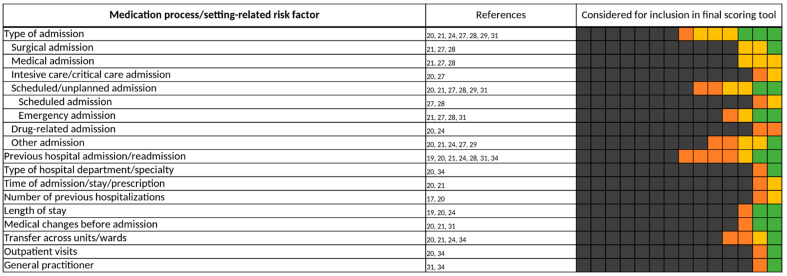
Medication process/setting-related risk factors. Each of the 14 boxes represents one of the included studies. The color of the boxes portrays whether a risk factor was not investigated in a study (gray), was investigated (orange), was significant in a first statistical analysis or based on expert consensus and therefore tested for inclusion in a final scoring tool (yellow), or whether a risk factor was included in a final scoring tool (green). The “citations”-column lists each study that at least investigated the respective risk factor.

For the medication process/setting-related risk factors (Figure 12), the type of admission was a commonly-viewed risk factor, being investigated in half of the included studies [20,21,24,27,28,29,31]. Here, authors differentiated between surgical, medical [21,27,28], or intensive care admissions [20,27], as well as scheduled or emergency admissions [20,21,27,28,29,31], and finally, whether the admission of a patient was drug-related [20,24]. However, the type of admission was often already predetermined by the study setting and therefore was not assessed. For example, O’Mahony et al. included only emergency admissions in their study and accordingly did not investigate the type of admission for inclusion in their predictive scoring tool [18].

When “type of admission” was included in a final scoring tool, emergency-admitted patients were considered to be at risk for the occurrence of a DRP most frequently [21,31]. Furthermore, previous hospital admission or re-admission were also examined in 7 of the 14 included studies [19,20,21,24,28,31,34]. The period in which the re-admission or the last admission had to take place differed throughout the studies from within 7 days [31,34] to over 3 months [31]. In two predictive scoring tools, “ART” and Hohl et al.’s clinical decision rules, “previous hospital admission/re-admission” was included as a risk factor for DRPs [31,34]. On the other hand, Nguyen et al. did include “previous hospital admission/re-admission” in their final scoring tool, but here it was considered to decrease the risk of the occurrence of a DRP [21].

## 4. Discussion

There is a great clinical need for the improved identification of patients at risk for DRP. The numerous predictive scoring tools proposed, so far, have already been the topic of excellent reviews [7,8,9,10,11,12,13], but to the best of our knowledge this is the first systematic review and analysis of scoring tools with a focus on the individual factors included in the tools. The heterogenicity previously noted at the level of the overall predictive scoring tools is matched by a comparable variability of individual risk factors as well as definitions of risk factors and thresholds of risk factors. Yet, in contrast to an assessment of overall scoring tools, where transferability was found to be limited, the focus on individual factors across multiple studies and their thematic classification may allow new approaches to identify possible risk score components for broader use.

We were able to identify and compile a total of 844 individual risk factors assessed as components of predictive scoring tools, which are all shown in Supplementary File S5. Since individual risk factors, as well as definitions and thresholds of risk factors assessed, varied broadly throughout the 14 studies, we chose to summarize these risk factors into categories to give a better understanding of the general types of risk factors while still providing a detailed look upon all investigated risk factors. Unlike previous reviews on this topic, we not only analyzed risk factors included in a final scoring tool but all risk factors that were considered and tested for inclusion independent of the statistical approach used by the authors. This offers a versatile insight into the selection process of risk factors for inclusion into a final scoring tool. Candidate risk factors frequently considered but rarely included in final scores may offer valuable insights and an indirect approach to overcome the typical “publication bias”. When a risk factor was assessed in one study but only found to be informative in one study, this factor can—with a grain of salt—be considered to have failed external validation.

Authors based their selection of candidate risk factors considered for inclusion in a final scoring tool either on literature research and/or expert consensus [26,34], on risk factor identification by statistical analysis of the patient’s characteristics [17,21,27,28,30], or both [18,19,20,22,24,29,31]. Unfortunately, if an expert consensus was chosen for the selection of the candidate risk factors [20,24,26,31,34], a reasoning for this selection was often not given by the authors [24,26,31].

We were also able to identify great heterogeneity regarding the study settings, outcome definitions, and statistical approaches used in the 14 studies (Table 1). This might have directly influenced whether a risk factor was finally included in a predictive scoring tool, leading to the often-varying results regarding the inclusion of a risk factor found in our analysis. It also argues for caution when interpreting negative findings for risk factors. In correspondence with the findings of previous reviews evaluating the performance of predictive scoring tools, this supports the common conception that some risk factors and predictive scoring tools at this stage may not be transferrable to more diverse settings [7,8,9,10,11,12,13].

As indicated in the methods, the terminology and definitions of DRP, ADE, ADR, and ME show considerable variation. Yu et al. and Bürkle et al. previously revealed great variation in the use of medication safety-related terms as well as great overlap in the functional meanings of the definitions of these terms [15,16]. Based on these findings, we deemed the investigation and comparison of risk factors appropriate, despite the stated different outcome definitions applied in the included studies. We found the terms, adverse drug event (ADE) [24,27,29,30,31,34] and adverse drug reaction (ADR) [17,18,19,22,31], to be most frequently applied as the outcome measurement in the 14 studies, while the other outcome definitions, drug-related problems (DRP) [20,28] or medication errors (ME) [21,26,34], were used to a lesser extent. In this respect, further studies using consistent definitions for outcome measurements like DRP, ME, ADE, and ADR, as well as standardized thresholds and definitions of risk factors, are needed to improve the transferability of predictive scoring tools. Notably, the recent definitions of medication safety-related terms given in the guidelines for good pharmacovigilance practices of the European Medicines Agency (EMA) [48] can be considered a first step in the right direction, thereby leading to a uniform application of these definitions and helping to resolve this issue of inconsistency in future studies.

Additionally, some risk factors may not have been assessed by an author in the first place because, instead, a corresponding factor from a different risk factor category was assessed. For example, Onder et al. and Lima et al. only investigated “diabetes mellitus” of the diagnosis-related risk factors, but not “drugs used in diabetes (ATC A10)” of the drug-related risk factors [17,22]. On the other hand, Bos et al., Geeson et al., and Nguyen et al. investigated only “drugs used in diabetes (ATC A10)” instead of “diabetes mellitus” [20,21,29]. This might have led to an underrepresentation of a risk factor. However, Falconer et al., Hohl et al., Sakuma et al., Tangiisuran et al., and Trivalle et al. investigated both “diabetes mellitus” as a diagnosis-related risk factor as well as “drugs used in diabetes (ATC A10)” as the corresponding drug-related risk factor [19,24,27,30,31,34]. Moreover, this also raises issues of the multicollinearity affecting the typical multivariable regression models as an explanation for clinically-plausible factors not being included in final scores.

Depending on the type of a scoring tool, some risk factors have to be met before the following risk factors of the scoring tool are evaluated. In Hohl et al.’s clinical decision rules, a patient must either have more than one comorbid condition or they had to be on antibiotics in the past 7 days before the other included risk factors are taken into account, valuing these two risk factors higher than the following [31]. Furthermore, some authors included the same risk factor more than once in their predictive scoring tool, but they used different thresholds for that factor and valued them in correspondence with their strength of effect on risk [22,26,30]. We recorded each of these risk factors as individual risk factors and included them in our analysis, but we did not incorporate whether a threshold of a risk factor or a risk factor itself was valued higher than others, except for risk factors that were considered to not increase the risk of DRPs. As stated before, we recorded these risk factors only as “investigated by the authors”, even though they were included in a final scoring tool, because we focused exclusively on risk factors that increase the risk of a DRP in this review.

Given the heterogenicity of the combinations of risk factors included in risk scores as well as the variation of the definitions of the risk factors between the studies, one might be tempted to consider efforts to construct a risk score from the published literature futile. However, as stated above, the rationale of the frequency of assessment and inclusion into a final predictive scoring tool may offer some indication of the external validity of an individual risk factor. Here, the graphical coding of the risk factor assessments provided in the figures (i.e., frequently assessed and frequently included into a score) may offer a crude but nonetheless robust estimate.

### 4.1. Five Most Investigated and Included Risk Factors

**Figure 13:**
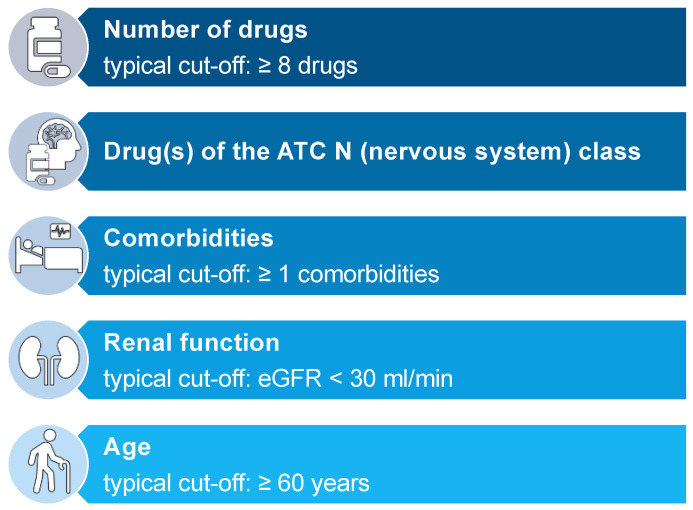
Five most frequently investigated risk factors and their respective typical cut-off values.

Using a new methodological approach to assess a large number of individual components of published predictive risk scoring tools rather than the overall risk scores, we were able to assemble five repeatedly assessed and confirmed risk factors. These risk factors may be broadly applicable to identify patients at risk for DRP in a hospitalized patient’s setting. Additionally, we attempted to give defined thresholds for these risk factors based on the analysis of the various thresholds investigated by the authors and included in their respective final scoring tool (Figure 13). These findings may serve as a basis for researchers working on novel predictive scoring tools. On top of this, the simple survey of the five risk factors allows their application as a rule of thumb to identify patients at risk for DRP by physicians in a clinical setting without an established predictive risk assessment tool.

Our analysis of the risk factor, “number of drugs”, revealed that over 50% of the authors who included “number of drugs” as a risk factor in their respective scoring tool considered a number above eight drugs to increase the risk of drug-related problems (Figure 5). This number is consistent with findings in one of our own previous studies where we were able to identify a median of 8 medications in 45,809 patients above the age of 70 years in 44 geriatric units in Bavaria, Germany. Of these patients, taking 8 drugs resulted in more than 20% taking at least one potentially inadequate medication (PIM) [49]. A higher number of drugs taken by the patient as well as potentially inadequate medications among those drugs go along with a high risk for interactions, therefore increasing the risk for adverse events in patients [50,51].

Drugs of the ATC N (nervous system) class were investigated in 11 of the 14 studies [17,19,20,21,24,27,28,29,30,31,34] and included in 6 final scoring tools [20,21,24,29,34]. The ATC N class contains anti-epileptics, antipsychotics, and antidepressants, alongside opioids. These drugs are usually considered to be “dirty drugs” since they interact with a broad spectrum of different receptors, thereby causing an equally broad spectrum of different adverse reactions in patients. Additionally, many of these drugs come with a high interaction potential and a narrow therapeutic index, increasing the risk of adverse events even further. For that reason, many drugs of the ATC N class are considered potentially inappropriate medication (PIM) and therefore included in tools for the identification of PIM in elderly patients like START/STOPP [52], PRISCUS [53], and FORTA [54]. Accordingly, the assessment of drugs of the ATC N class for inclusion in a predictive scoring tool is reasonable. On the contrary, though frequently investigated, drugs of the ATC L and ATC M class were rarely included in a final scoring tool. ATC L contains chemotherapeutics and immunosuppressants. These drugs are also considered to have a narrow therapeutic index, often interfere with other drugs, as well as causing reduced liver or renal function of the patient, and can therefore lead to adverse drug reactions, rightfully justifying the assessment of this drug class as a risk factor for DRPs. On the other hand, patients receiving these drugs are usually monitored more closely, enabling physicians to identify and adjust the medication before the occurrence of an ADR. This could explain the rare inclusion of this drug class into a final scoring tool. Given the recent advances and changes in therapeutic patterns in immune disease and in oncology (including the steep increase in the availability and use of numerous new oral anticancer drugs), new drug classes of interest for risk scoring may soon be identified.

Comorbidities were often investigated for inclusion as a risk factor in a final scoring tool but were assessed differently by the authors throughout the 14 studies [17,18,19,20,22,24,26,28,30,31]. Of the specific diseases, diseases of the nervous and mental system [17,18,19,20,22,24,27,28,30,31,34], diseases of the circulatory system [17,18,19,20,22,24,27,28,30,31,34], and diseases of the endocrine system [17,19,20,22,24,27,28,30,31,34] were investigated most frequently. However, we found great disagreement with the authors’ findings about which specific disease should be included in a final scoring tool. Congestive heart failure [17,18,19,22,24,27,31,34] and diabetes mellitus [17,19,22,24,27,30,31,34] were both commonly investigated, each in 8 of the 14 studies, but were only included in 2 final scoring tools, respectively [17,22,34]. Likewise, dementia was assessed as a risk factor for DRPs in 8 of the 14 studies [17,18,19,20,22,24,27,30], but was included even less, with only 1 inclusion in a final scoring tool [27]. Based on these findings, we can conclude that comorbidities as a whole, rather than a specific disease, might be more relevant to estimate the risk of the occurrence of a DRP in patients. Unfortunately, again authors were divided over the way of assessment of comorbidities, presenting the number of comorbidities [18,19,20,22,24,26,30,31] and the Charlson Comorbidity Index (CCI) [17,20,26,28] as the most applied methods.

Renal function was investigated in 12 of the 14 studies [17,18,19,20,22,24,26,27,28,29,31,34]. Here, 9 of the 14 studies were using the GFR as the way to record renal function [18,19,20,22,24,26,28,29,34]. When included in a final scoring tool, thresholds for the GFR were inhomogeneous. While Onder et al. considered a GFR below 60 mL/min to already increase the risk of a DRP in patients [22], O’Mahony et al. used a narrower threshold with a GFR below 30 mL/min [18]. Saedder et al. included both thresholds in their respective scoring tool, but a GFR below 30 mL/min was valued higher than a GFR below 60–30 mL/min [26]. Based on these results, we are not able to give a defined threshold for the GFR to assess in future scoring tools. Yet, it is well known that a GFR below 30 mL/min might interfere with the elimination of drugs more commonly than a GFR below 60–30 mL/min [55,56].

Finally, patients’ age was investigated in all 14 studies included in this review [17,18,19,20,21,22,24,26,27,28,29,30,31,34]. The thresholds for patients’ age differed broadly throughout the studies (Figure 11). Furthermore, O’Mahony et al., Onder et al., Tangiisuran et al., and Trivalle et al. only included patients of at least 65 years of age in their respective study, predetermining the thresholds for patients’ age as a risk factor (Table 1). Our analysis revealed that most authors considered patients of at least 60 years of age at risk for the occurrence of a DRP. Even though all authors of the 14 studies investigated “age” as a risk factor, it was only included in 6 of the 14 final scoring tools [18,21,28,29,31,34]. It is well established that a higher age is associated with a decrease in the GFR of an individual, a higher burden of comorbidities, and therefore a higher prescription of drugs [57,58]. Altogether, this increases the risk for adverse events, making age a reasonable risk factor to be considered for inclusion in a final predictive scoring tool. On the other hand, this correlation might also cause an insignificant result for “age” as an independent risk factor in statistical methods like multivariate logistic regression analysis, possibly adding no further benefit in performance by including patients’ age in a final scoring tool. This possible multicollinearity should be taken into account while considering “age” as a risk factor.

Besides the stated frequently assessed risk factors, we, however, also found risk factors that were rarely investigated. These especially include risk factors of the categories, “vital sign-related risk factors”, “patient-related risk factors”, and “medication process/setting-related risk factors” (Figure 9, Figure 10 and Figure 12). To evaluate the importance of these risk factors properly, further studies are needed to improve data quality.

### 4.2. Limitations

In this systematic review, we only investigated risk factors that were considered for inclusion or were included in a predictive scoring tool. Consequently, risk factors that were not considered for use in a predictive scoring tool were therefore not examined in detail. This might cause some individual risk factors that have been reviewed in other studies to not be assessed in this review. In addition, the gathering of information on all considered risk factors as well as the results of the statistical approaches for each risk factor was sometimes difficult, as data were not available in the included studies, their respective supplement material or references, or was not provided in other ways by the authors. Furthermore, due to the enormous amount of individual risk factors and different definitions as well as thresholds of these risk factors, the assignment of risk factors to an appropriate risk factor category for presentation reasons was necessary but not always decisive. However, to minimize subjectivity, information was reviewed by at least three of the authors (L.J.-P., H.F.N., B.P., and/or A.R.) in cases of uncertainty and then applied, to the best of our knowledge and understanding, according to our developed presentation scheme (Figure 1). To further improve the quality of the review process, this systematic review was conducted according to the Preferred Reporting Items for Systematic Reviews and Meta-Analyses (PRISMA).

## 5. Conclusions

Plenty of predictive scoring tools for the identification of patients at risk for drug-related problems have been proposed in recent years to improve patient safety by helping to better target attention and specialists’ resources to those patients most likely to benefit. However, the transferability of these scoring tools from their respective original study setting to a more generalized setting has already been found to be limited in previous reviews.

In this review we took a different approach and assessed the individual components (i.e., items) that make up these scores in different combinations. In contrast to the overall predictive scoring tools (with their missing or rather limited external validation), many individual items have been included in a number of studies and thus have undergone a much more extensive external validation than the final scores, in which they were included in various combinations. Thus, instead, this systematic review focused on the individual risk factors considered and included in multiple studies and their thematic classification, as it may allow new approaches to identify possible risk score components of broader use.

We were able to identify five easily ascertainable risk factors that may aid the identification of patients at risk for DRP irrespective of the existence of an underlying scoring system. On the other hand, the present approach also allowed the identification of candidate risk factors repeatedly found unsuitable for risk assessment, which may help to save resources in future studies. Finally, the technically simple yet informative approach to constructing a surrogate external validation based on numbers of studies assessing and actually including a risk factor into a predictive scoring tool may have very practical implications beyond the topic of the present review. It is easily conceivable that this approach may be applicable more broadly to overcome the limited or missing external validation of risk score components in general. Therefore, we encourage the application of our approach and its assessment in other types of predictive risk scoring tools comprised of multiple individual risk factors.

Taken together, our findings may act as a guide for the selection of candidate risk factors in future scoring tools or for the refinement of existing scores undergoing the validation process. For instance, we are currently validating our own predictive risk score for the identification of high-risk patients at the hospital in Fürth, Germany.

## Figures and Tables

**Table 1:** Characteristics of the 14 included studies.

**Author, Year**	**Name of Scoring Tool**				**Country**	**Study Design**
**Bos, J.M.** 2018 [29]	no specific name				The Netherlands	prospective
**Falconer, N.** 2014 [34]	ART (Assessment of Risk Tool)				New Zealand	n.a. (practice report)
**Falconer, N.** 2020 [24]	AIME (Adverse Inpatient Medication Event model)				Australia	retrospective
**Geeson, C.** 2019 [20]	MOAT (Medicines Optimisation Assessment Tool)				United Kingdom	prospective
**Hohl, C.M.** 2012 [31]	(1) ADR CDR (2) ADE CDR				Canada	prospective
**Lima, S.** 2020 [17]	no specific name				Brazil	retrospective
**Nguyen, T.-L**. 2017 [21]	PRISMOR (Predicting In-hospital Significant Medication errors)				France	prospective
**O’Mahony, D.** 2018 [18]	ADRROP (ADR Risk in Older People)				Ireland	retrospective and prospective
**Onder, G**. 2010 [22]	GerontoNet ADR Risk Score				Italy, Belgium, United Kingdom, the Netherlands	retrospective and prospective
**Saedder, E.** 2016 [26]	MERIS (Medicine Risk Score)				Denmark	retrospective and prospective
**Sakuma, M.** 2012 [27]	no specific name				Japan	prospective
**Tangiisuran, B.** 2014 [19]	BADRI (Brighton Adverse Drug Reactions Risk model)				United Kingdom, Italy, Belgium, the Netherlands	prospective
**Trivalle, C.** 2011 [30]	no specific name				France	prospective
**Urbina, O.** 2014 [28]	no specific name				Spain	prospective
**Author, Year**	**Setting and Population**				**Sample Size**	
**Bos, J.M.** 2018 [29]	patients admitted to the surgical, urological, and orthopedic ward [29,35]				6780 admissions corresponding to 5940 patients (200 bootstrap samples for internal validation)	
**Falconer, N.** 2014 [34]	n.a. (practice report)				n.a. (practice report)	
**Falconer, N.** 2020 [24]	adult patients admitted to the general medical and/or the Geriatric Assessment and Rehabilitation Unit [24]				1982 (data were split into deciles: model development in 9 out of 10 parts and validation in 1 out of 10 parts. Repetition of the process 200 times.)	
**Geeson, C.** 2019 [20]	patients (≥18 years old) admitted to medical wards: general, emergency, elderly medicine [20,36]				1503 admissions corresponding to 1444 patients (200 bootstrap samples for internal validation.)	
**Hohl, C.M.** 2012 [31]	patients (>18 years old) presenting to the emergency department [31]				1591	
**Lima, S.** 2020 [17]	patients (>18 years old) admitted to specific departments: neurology, mental health, nephrology, urology, cardiology, oncology (not receiving chemotherapy), gastroenterology, rheumatology, surgery [17]				343 occurrences of ADR 686 matched controls Total: 1029 (development sample: 2/3 of the cases and the respective controls; validation sample: 1/3)	
**Nguyen, T.-L.** 2017 [21]	patients (≥18 years old) admitted to hospital [21]				1408 (500 bootstrap samples for internal validation)	
**O’Mahony, D.** 2018 [18]	(1) patients (≥65 years old) admitted to the general medical and surgical services (emergency admissions) [18,37] (2) patients (≥65 years old) admitted to medical and surgical services (emergency admissions) [18,38] (3) patients (≥65 years old) (emergency admissions) [18,39] (4) patients (≥65 years old) admitted to medical and surgical services (emergency admissions) [18,40]				513 (1) 600 (2) 732 (3) 372 (4) Total: 2217 (derivation set: 1687; validation set: 530)	
**Onder, G.** 2010 [22]	(1) patients (≥65 years old) from GIFA sample [22] (2) patients (≥65 years old) admitted to geriatric and internal medicine wards [22]				5936 (1) (development set) 483 (2) (validation set)	
**Saedder, E.** 2016 [26]	(1) historic patients: patients (≥65 years old), orthopedic ward [26,32] (2) historic patients: patients (≥70 years old), internal medicine [26,33] (3) recent patients: patients (≥18 years old), medical admission unit (endocrinology, respiratory medicine, gastroenterology, hepatology, cardiology) [26] (4) prospective pilot study: patients (≥18 years old), acute admission unit [26]				53 (1) 50 (2) 146 (3) Total: 249 (modeling set) 53 (4) (validation set)	
**Sakuma, M.** 2012 [27]	patients (≥15 years old) admitted to medical and surgical wards and intensive care units [27,41]				1729 (derivation set) 1730 (validation set) Total: 3459	
**Tangiisuran, B.** 2014 [19]	(1) patients (≥65 years old) admitted to elderly care and stroke wards (elderly care wards only accept patients ≥ 80 years old) [19,42] (2) patients (≥65 years old) admitted to geriatric and internal medicine wards [19,22]				690 (1) (development set) 483 (2) (validation set)	
**Trivalle, C.** 2011 [30]	patients (≥65 years old) who experienced an ADE during 4 weeks of the study period in geriatric rehabilitation centers [30,43]				576 (bootstrapping for internal validation)	
**Urbina, O.** 2014 [28]	patients (>18 years old) admitted to surgical and medical wards [28]				8713 admissions corresponding to 7202 patients (training set) 4058 admissions corresponding to 3598 patients (validation set)	
		**Identification/Evaluation of Individual Risk Factors**				
**Author, Year**	**Number of Risk Factors Included in the Final Scoring Tool**	**Literature ** **Review/Search**	**(Expert)** **Consensus**	**Statistical Method**	**Internal ** **Validation**	**External Validation**
**Bos, J.M.** 2018 [29]	5	✓	✗	✓	✓	
**Falconer, N.** 2014 [34]	38	✓	✓	✗	✗	Falconer et al., 2017 [44]
**Falconer, N.** 2020 [24]	10	✓	✓	✓	✓	
**Geeson, C.** 2019 [20]	11	✓	✓	✓	✓	
**Hohl, C.M.** 2012 [31]	5 (1) 8 (2)	✓	✓	✓	✓	Hohl et al., 2018 [45]
**Lima, S.** 2020 [17]	14	✗	✗	✓	✓	
**Nguyen, T.-L.** 2017 [21]	11	✗	✗	✓	✓	
**O’Mahony, D.** 2018 [18]	9	✓	✗	✓	✓	
**Onder, G.** 2010 [22]	6	✓	✗	✓	✗	Onder et al., 2010 [22] O’Connor et al., 2012 [37] Petrovic et al., 2017 [25]
**Saedder, E.** 2016 [26]	3	✓	✓	✗	✓	Høj et al., 2021 [46]
**Sakuma, M.** 2012 [27]	8	✗	✗	✓	✓	
**Tangiisuran, B.** 2014 [19]	5	✓	✗	✓	✓	Tangiisuran et al., 2014 [19]
**Trivalle, C.** 2011 [30]	3	✗	✗	✓	✓	
**Urbina, O.** 2014 [28]	14	✗	✗	✓	✓	Ferrández et al., 2018 [47]

ADE, adverse drug event; ADR, adverse drug reaction; CDR, clinical decision rule; GIFA, Gruppo Italiano di Farmacoepidemiologia nell’Anziano (Italian Group of Pharmacoepidemiology in the Elderly); n.a., not available.

## Data Availability

The data presented in this study are openly available in this article and in Supplementary File S5.

## Supplementary Materials

The following supporting information can be downloaded at: https://www.mdpi.com/article/10.3390/jcm11175185/s1, Supplementary File S1: Review Protocol; Supplementary File S2: Authors’ outcome measurements and definitions; Supplementary File S3: Inclusion & Exclusion Criteria; Supplementary File S4: Search Strategy; Supplementary File S5: Detailed presentation of all risk factors; Supplementary File S6: PRISMA 2020 for Abstracts Checklist; Supplementary File S7: PRISMA 2020 Checklist. References [59,60,61,62,63,64,65,66,67,68,69,70,71,72] are cited in the Supplementary File S2.
