# Supplementary material for: Systematic Review of Risk Factors Assessed in Predictive Scoring Tools for Drug-Related Problems in Inpatients
Source: J Clin Med. 2022 Sep 1;11(17):5185. doi: 10.3390/jcm11175185 (PMC9457151; doi:10.3390/jcm11175185)
Supplement: Supplementary file 1 [file jcm-11-05185-s001.zip › Supplementary File S1.pdf]

Protocol of
Systematic Review of Risk Factors Assessed in Predictive Scoring Tools for Drug-related Problems in Inpatients

Citation
Jung-Poppe, L.; Nicolaus, H.F.; Roggenhofer, A.; Altenbuchner, A.; Dormann, H.; Pfistermeister, B. and Maas, R. Systematic Review of Risk Factors Assessed in Predicitve Scoring Tools for Drug-related Problems in Inpatients

Review question
Which individual risk factors were evaluated for risk assessment and which were finally included as components of risk scores in published predictive scoring tools for the detection of inpatients at risk for drug-related problems (DRP)?

Searches
A literature search will be performed in 3 different databases: National Library of Medicine PubMed®, Cochrane Library and Scopus.
The search strategy will combine Medical Subject Headings (MESH) terms (PubMed®) and free text search with Boolean operators. A filter for the period of time comprising the set inclusion time period of January 2011 to August 2021 will be used for all three databases. In addition, a hand search in Google will be conducted as well as a hand search of the references of all studies that meet the inclusion and exclusion criteria.

Types of studies to be included
All original studies that evaluate and discuss the development of a general predictive scoring tool for identification of patients at risk for DRP in an clinical inpatient setting will be included if they meet the inclusion criteria.

Condition or domain being studied
Individual components of published risk scores (i.e. risk factors) developed in clinical inpatient settings that contribute to the identification of patients with a high risk of future drug-related problems (DRP) [comprised of adverse drug events (ADE) and/or adverse drug reactions (ADR) and medication errors (ME)] As definitions of DRP, ADR and ME studies may vary between studies common denominators for definitions will have to be defined.

Participants/population
Hospitalized adult patients

Inclusion criteria
- English/German language
- Original articles / practice reports
- Access to full text
- Period of time from 01/2011 to 08/2021
- Inpatient setting
- Adult patients
- Articles investigating medication errors made by physicians
- Predictive scoring tools applied by pharmacists/physicians or by an automatic/electronic assessment

- General predictive scoring tools for identification of patients at risk for drug-related problems which are applicable in the moment of hospitalization/ during hospitalization
- Outcome: Drug-related problems (including medication errors, adverse drug reactions/events)

Exclusion criteria:
- Other languages than English or German
- Other article types (e.g., reviews, meta-analyses, editorials, summary articles, case reports)
- Full text could not be located
- Publication before 01/2011 and after 08/2021
- Outpatient setting (e.g., ambulatory care, primary health care, nursing homes)
- Underaged patients (according to the legal system of the respective country)
- Articles investigating medication errors made by nurses
- Predictive scoring tools applied by patients themselves
- Articles only identifying ADE/ADR/ME or even risk factors for drug-related problems but without developing a predictive scoring tool
- Scoring tools developed for other purposes than identification of patients at risk for DRP
- Predictive scoring tools focusing only on
    - A specific (narrow) demographic patient population (except for geriatric patients)
    - Patients with specific conditions
    - A single step in the medication process
    - A specific DRP
- Other outcomes:
    - Inappropriate prescribing
    - Underprescribing
    - Focusing only on a specific DRP
    - Medication discrepancies
    - Medication administration errors
    - General adverse events/ harm not caused by drugs
    - ADR-related hospital (re-)admissions
    - DRP at/after discharge from hospital

Main outcome(s)

Frequency of assessment of an individual risk factor as a possible component for inclusion into a risk score across different published risk scores (a) and frequency of inclusion as significantly contribution component into a published risk score (b). Unless stated otherwise, a statistically significant association of the individual components of a risk score with future DRP (ADR, ADE, ME) will be assumed for (b).

Additional outcome(s)

The selection process of individual risk factors considered for the inclusion in a predictive scoring tool as well as the performance of individual risk factors to detect inpatients at risk for DRP and their inclusion in a final scoring tool to offer a new approach to arrive at validated and more universally applicable components for a risk score.

Data extraction (selection and coding)

A detailed information on the literature search strategy for each database will be saved as a separate file. Records identified by each conducted search will be screened for review articles, duplicates, and articles in other languages than English or German and removed. All remaining records will be screened by title. Appropriate records with a title suggesting its relevance to the topic (development of a predictive scoring tool) will then be screened by abstract for an association with risk factors for DRP. Subsequently, full texts of the reports retrieved for eligibility will be evaluated using the prespecified

in- and exclusion criteria. Full text evaluation of the records identified from citation search and hand search in Google will be undertaken accordingly. Two authors (L.J-P. and H.F.N.) will review all studies retrieved for eligibility independently to ensure a valid and reproducible process.
Data items extracted from the studies will include:
Fist author, year of publication, name of the predictive scoring tool, country, study design, study population and setting, sample size, outcome, number of risk factors in the scoring tool, method of identification of risk factors, model development and performance, internal and external validation and all risk factors considered for inclusion.
Excel spreadsheets will be used to record the extracted data.

Risk of bias (quality) assessment

To minimize selection bias, the review of all studies retrieved for eligibility will be performed individually by two authors (L.J-P. and H.F.N.). To minimize bias because of missing information regarding the extracted data items, corresponding authors will be contacted directly in cases of unclear or missing information. In case of discrepancies between the two reviewers or uncertainty a third review author will be consulted for a final decision.

Strategy for data synthesis

Because identified studies are expected to be inhomogeneous in terms of study design, statistical approaches and outcome and risk factor definitions, a narrative review will be executed in text and tables to present the results. A qualitative assessment of the study characteristics and development of the respective predictive scoring tool will allow comparisons between the included studies to be made. All risk factors named in the included publications will be listed regardless of inclusion in the final predictive scoring tool. These risk factors are to be classified in 6 dimensions: Drug-related risk factors, diagnosis-related risk factors, laboratory value-related risk factors, vital sign-related risk factors, patient-related risk factors and medication process/hospital setting-related risk factors. Each identified risk factor will be evaluated on whether the risk factor has been investigated, whether the risk factor has been significant in the statistical analysis applied by the authors and whether the risk factor has been included the final scoring tool. Quantitative analysis of particularly common risk factors and thresholds used for risk factors may offer an indication of which factors are strongly predictive and might serve as a basis for the development of new predictive scoring tools.

Analysis of subgroups or subsets

Subcategories of risk factors or cut off values / thresholds of continuous factors.

Contact details for further information

Lea Jung-Poppe
lea.jung-poppe@fau.de

Prof. Dr. Renke Maas
renke.maas@fau.de

Organisational affiliation of the review

Friedrich-Alexander-Universität Erlangen-Nürnberg
Institute of Experimental and Clinical Pharmacology and Toxicology, Erlangen, Germany
https://www.pharmakologie.med.fau.de/institut/lehrstuhl-fuer-klinische-pharmakologie-und-klinische-toxikologie/

Review team members and their organisational affiliations

Lea Jung-Poppe. Friedrich-Alexander-Universität Erlangen-Nürnberg, Institute of Experimental and Clinical Pharmacology and Toxicology, Erlangen, Germany.

Hagen Fabian Nicolaus. Friedrich-Alexander-Universität Erlangen-Nürnberg, Institute of Experimental and Clinical Pharmacology and Toxicology, Erlangen, Germany and University Hospital Erlangen, Erlangen, Germany.
Anna Roggenhofer. Friedrich-Alexander-Universität Erlangen-Nürnberg, Institute of Experimental and Clinical Pharmacology and Toxicology, Erlangen, Germany.
Anna Altenbuchner. Friedrich-Alexander-Universität Erlangen-Nürnberg, Institute of Experimental and Clinical Pharmacology and Toxicology, Erlangen, Germany.
Prof. Dr. med. Harald Dormann. Fürth Hospital, Central Emergency Department, Fürth, Germany.
Dr. rer. nat. Barbara Pfistermeister. Fürth Hospital, Hospital Pharmacy, Fürth, Germany.
Prof. Dr. med. Renke Maas. Friedrich-Alexander-Universität Erlangen-Nürnberg, Institute of Experimental and Clinical Pharmacology and Toxicology, Erlangen, Germany.

Type and method of review
Systematic review

Anticipated or actual start date
1st February 2021

Anticipated completion date
15th June 2022

Funding sources/sponsors
This research is funded in part by the German Federal Ministry of Health (Bundesministerium für Gesundheit), grant number ZMVI1-2519ATS004Z. We acknowledge financial support by Deutsche Forschungsgemeinschaft and Friedrich-Alexander-Universität Erlangen-Nürnberg within the funding program "Open Access Publication Funding".

Conflicts of interest
The authors declare no conflict of interest. The funders had no role in the design of the study.

Language
English

Country
Germany
