# Supplementary material for: Systematic Review of Risk Factors Assessed in Predictive Scoring Tools for Drug-Related Problems in Inpatients
Source: J Clin Med. 2022 Sep 1;11(17):5185. doi: 10.3390/jcm11175185 (PMC9457151; doi:10.3390/jcm11175185)
Supplement: Supplementary file 1 [file jcm-11-05185-s001.zip › Supplementary File S2.pdf]

Supplementary File S2 – Authors´ outcome measurements and definitions

Author, Year	Outcome measurement	Outcome definition
**Bos, J.M.** 2018 [29]	ADE	Clinically relevant, potentially preventable adverse drug events that led to death, temporary or permanent disability, increased length of hospital stay, or readmission within 30 days [29,35].
**Falconer, N.** 2014 [34]	ADE, ME	n.a.
**Falconer, N.** 2020 [24]	ADE	"An appreciably harmful or unpleasant reaction, resulting from an intervention related to the use of a medicinal product; adverse effects usually predict hazard from future administration and warrant prevention, or specific treatment, or alteration of the dosage regimen, or withdrawal of the product." [59,60]. This included any negative inpatient outcome or injury resulting in clinical signs, symptoms or physiological abnormalities, related to the use of a medication during hospitalization [24].
**Geeson, C.** 2019 [20]	DRP	The occurrence of at least one moderate or severe preventable MRP. MRPs were defined as "all circumstances involving a patient's drug treatment that actually, or potentially, interfere with the achievement of an optimal outcome" [20,61].
**Hohl, C. M.** 2012 [31]	ADR (1)<br>ADE (2)	(1) An adverse drug reaction was "a response to a drug that is noxious and unintended and occurs at doses normally used in man for the prophylaxis, diagnosis, or therapy of disease." [31,62]<br>(2) An ADE was defined as an "untoward and unintended event arising from the use of prescription or over-the-counter medications." [31,59,62,63]
**Lima, S.** 2020 [17]	ADR	Adverse drug reaction was defined as a response to a medicine which is noxious and unintended, and which occurs at doses normally used in man for the prophylaxis, diagnosis, or therapy of disease, or for the modification of a physiological function. According to this definition, non-adherence to the treatment, overdose either accidental or intentional, treatment failure and administration errors are not considered ADR [17,64].
**Nguyen, T.-L.** 2017 [21]	ME	At least one clinically significant ME at any time during in-hospital stay. MEs were defined as either unintentional medication discrepancies (i.e., involuntary divergence between admission prescription and best possible medication history obtained from a minimum of 3 sources of information) or prescribing errors (i.e., non-adherence to recognized clinical prescribing guidelines) [21]
**O'Mahony, D.** 2018 [18]	ADR	ADRs were ascertained according to the WHO ADR definition, i.e., a response to a drug that is noxious and unintended and that occurs at doses normally used in man for prophylaxis, diagnosis or therapy of disease or the modification of physiological function [18,65].
**Onder, G.** 2010 [22]	ADR	An ADR was defined as any noxious, unintended, and undesired effect of a drug, excluding therapeutic failures, intentional and accidental poisoning, and drug abuse [22,62]
**Saedder, E.** 2016 [26]	ME	Definition by Lisby et al. who define MEs as "errors in the stages of the medication process – ordering, dispensing, administering and monitoring the effect – causing harm or implying a risk of harming the patient" [26,66]. This definition requires actual harm or a risk of harming the patient. Harm was defined according to the WHO seriousness criteria, which is "A serious adverse reaction corresponds to any untoward medical occurrence that at any dose results in death, is life-threatening, requires inpatient hospitalization or prolongation of existing hospitalization, results in persistent or significant disability or incapacity, is a congenital anomaly/birth defect" [26,67,68].
**Sakuma, M.** 2012 [27]	ADE	ADE is an injury due to a medication use regardless of existence of MEs [41,69]
**Tangiisuran, B.** 2014 [19]	ADR	Definition of an ADR by Edwards and Aronson: "an appreciably harmful or unpleasant reaction, resulting from an intervention related to the use of a medicinal product, which predicts hazard from future administration and warrants prevention or specific treatment, or alteration of the dosage regimen, or withdrawal of the product" [19,59].
**Trivalle, C.** 2011 [30]	ADE	A sign or symptom caused by one drug or a drug combination was considered a ''probable'' adverse effect if: (i) the temporal sequence between the beginning of the drug combination and the symptom was reasonable, (ii) there was a known response pattern, (iii) the signs or symptoms were improved by discontinuation of the drugs, (iv) the signs and symptoms could not reasonably be explained by the characteristics of the patient's clinical condition [30,70].

**Urbina, O.** 2014 [28]	DRP	Drug-related problems are any circumstance involving drug therapy that actually or potentially interferes with an optimal outcome. Medication error is any preventable event that may cause or lead to inappropriate medication use or patient harm while the medication is in the control of the healthcare professional, patient, or consumer [28,71]. Adverse drug reaction is a response to a drug that is noxious and unintended and occurs at doses normally used in man for the prophylaxis, diagnosis, or therapy of disease or for modification of physiological function [28,64]. Adverse drug event is injury resulting from medical intervention related to a drug [28,63,72]. To allow a distinction from adverse events in general, a causal link to a drug effect is required [16,28,63].

ADE, adverse drug event; ADR, adverse drug reaction; DRP, drug-related problems; ME, medication error; MRP, medication-related problems; n.a., not available; WHO, World Health Organization.

References from main text

16. Bürkle, T.; Müller, F.; Patapovas, A.; Sonst, A.; Pfistermeister, B.; Plank-Kiegele, B.; Dormann, H.; Maas, R. A new approach to identify, classify and count drug-related events. *British Journal of Clinical Pharmacology* **2013,** *76*, 56-68. doi:10.1111/bcp.12189.
17. Lima, S. I. V. C.; Martins, R. R.; Saldanha, V.; Silbiger, V. N.; Dos Santos, I. C. C.; Araújo, I. B. D.; Oliveira, A. G. Development and validation of a clinical instrument to predict risk of an adverse drug reactions in hospitalized patients. *PLOS ONE* **2020,** *15* (12), e0243714. doi:10.1371/journal.pone.0243714.
18. O'Mahony, D.; O'Connor, M. N.; Eustace, J.; Byrne, S.; Petrovic, M.; Gallagher, P. The adverse drug reaction risk in older persons (ADRROP) prediction scale: derivation and prospective validation of an ADR risk assessment tool in older multi-morbid patients. *European Geriatric Medicine* **2018,** *9* (2), 191-199. doi:10.1007/s41999-018-0030-x.
19. Tangiisuran, B.; Scutt, G.; Stevenson, J.; Wright, J.; Onder, G.; Petrovic, M.; Van Der Cammen, T. J.; Rajkumar, C.; Davies, G. Development and Validation of a Risk Model for Predicting Adverse Drug Reactions in Older People during Hospital Stay: Brighton Adverse Drug Reactions Risk (BADRI) Model. *PLoS ONE* **2014,** *9* (10), e111254. doi:10.1371/journal.pone.0111254.
20. Geeson, C.; Wei, L.; Franklin, B. D. Development and performance evaluation of the Medicines Optimisation Assessment Tool (MOAT): a prognostic model to target hospital pharmacists' input to prevent medication-related problems. *BMJ Quality & Safety* **2019,** *28* (8), 645-656. doi:10.1136/bmjqs-2018-008335.
21. Nguyen, T.-L.; Leguelinel-Blache, G.; Kinowski, J.-M.; Roux-Marson, C.; Rougier, M.; Spence, J.; Le Manach, Y.; Landais, P. Improving medication safety: Development and impact of a multivariate model-based strategy to target high-risk patients. *PLOS ONE* **2017,** *12* (2), e0171995. doi:10.1371/journal.pone.0171995.
22. Onder, G.; Petrovic, M.; Tangiisuran, B.; Meinardi, M. C.; Markito-Notenboom, W. P.; Somers, A.; Rajkumar, C.; Bernabei, R.; Van Der Cammen, T. J. M. Development and Validation of a Score to Assess Risk of Adverse Drug Reactions Among In-Hospital Patients 65 Years or Older. *Archives of Internal Medicine* **2010,** *170* (13). doi:10.1001/archinternmed.2010.153.
24. Falconer, N.; Barras, M.; Abdel-Hafez, A.; Radburn, S.; Cottrell, N. Development and validation of the Adverse Inpatient Medication Event model (AIME). *British Journal of Clinical Pharmacology* **2020**. doi:10.1111/bcp.14560.
26. Saedder, E. A.; Lisby, M.; Nielsen, L. P.; Rungby, J.; Andersen, L. V.; Bonnerup, D. K.; Brock, B. Detection of Patients at High Risk of Medication Errors: Development and Validation of an Algorithm. *Basic & Clinical Pharmacology & Toxicology* **2016,** *118* (2), 143-149. doi:10.1111/bcpt.12473.
27. Sakuma, M.; Bates, D. W.; Morimoto, T. Clinical prediction rule to identify high-risk inpatients for adverse drug events: the JADE Study. *Pharmacoepidemiology and Drug Safety* **2012,** *21* (11), 1221-1226. doi:10.1002/pds.3331.
28. Urbina, O.; Ferrández, O.; Grau, S.; Luque, S.; Mojal, S.; Marin-Casino, M.; Mateu-de-Antonio, J.; Carmona, A.; Conde-Estévez, D.; Espona, M.; González, E.; Riu, M.; Salas, E. Design of a score to identify hospitalized patients at risk of drug-related problems. *Pharmacoepidemiology and Drug Safety* **2014,** *23* (9), 923-932. doi:10.1002/pds.3634.
29. Bos, J. M.; Kalkman, G. A.; Groenewoud, H.; Van Den Bemt, P. M. L. A.; De Smet, P. A. G. M.; Nagtegaal, J. E.; Wieringa, A.; Van Der Wilt, G. J.; Kramers, C. Prediction of clinically relevant adverse drug events in surgical patients. *PLOS ONE* **2018,** *13* (8), e0201645. doi:10.1371/journal.pone.0201645.
30. Trivalle, C.; Burlaud, A.; Ducimetière, P. Risk factors for adverse drug events in hospitalized elderly patients: A geriatric score. *European Geriatric Medicine* **2011,** *2* (5), 284-289. doi:10.1016/j.eurger.2011.07.002.

31. Hohl, C. M.; Yu, E.; Hunte, G. S.; Brubacher, J. R.; Hosseini, F.; Argent, C. P.; Chan, W. W. Y.; Wiens, M. O.; Sheps, S. B.; Singer, J. Clinical Decision Rules to Improve the Detection of Adverse Drug Events in Emergency Department Patients. *Academic Emergency Medicine* **2012,** *19* (6), 640-649. doi:10.1111/j.1553-2712.2012.01379.x.
34. Falconer, N.; Nand, S.; Liow, D.; Jackson, A.; Seddon, M. Development of an electronic patient prioritization tool for clinical pharmacist interventions. *American Journal of Health-System Pharmacy* **2014,** *71* (4), 311-320. doi:10.2146/ajhp130247.
35. Bos, J. M.; Van Den Bemt, P. M. L. A.; Kievit, W.; Pot, J. L. W.; Nagtegaal, J. E.; Wieringa, A.; Van Der Westerlaken, M. M. L.; Van Der Wilt, G. J.; De Smet, P. A. G. M.; Kramers, C. A multifaceted intervention to reduce drug-related complications in surgical patients. *British Journal of Clinical Pharmacology* **2017,** *83* (3), 664-677. doi:10.1111/bcp.13141.
41. Morimoto, T.; Sakuma, M.; Matsui, K.; Kuramoto, N.; Toshiro, J.; Murakami, J.; Fukui, T.; Saito, M.; Hiraide, A.; Bates, D. W. Incidence of Adverse Drug Events and Medication Errors in Japan: the JADE Study. *Journal of General Internal Medicine* **2011,** *26* (2), 148-153. doi:10.1007/s11606-010-1518-3.

Additional references in this supplementary file:

59. Edwards, I. R.; Aronson, J. K. Adverse drug reactions: definitions, diagnosis, and management. *Lancet* **2000,** *356* (9237), 1255-9. doi:10.1016/s0140-6736(00)02799-9.
60. Aronson, J. K.; Ferner, R. E. Clarification of Terminology in Drug Safety. *Drug Safety* **2005,** *28* (10), 851-870. doi:10.2165/00002018-200528100-00003.
61. Pharmaceutical Care Network Europe. The PCNE classification V 7.0. 2016. Available online: https://www.pcne.org/upload/files/152_PCNE_classification_V7-0.pdf (accessed on 27 July 2022).
62. World Health Organization. International drug monitoring: the role of the hospital. In *World Health Organization technical report series, no. 425,* Geneva, Switzerland, 1969. Available online: https://apps.who.int/iris/handle/10665/40747 (accessed on 27 July 2022).
63. Nebeker, J. R.; Barach, P.; Samore, M. H. Clarifying adverse drug events: a clinician's guide to terminology, documentation, and reporting. *Ann Intern Med* **2004,** *140* (10), 795-801. doi:10.7326/0003-4819-140-10-200405180-00009.
64. World Health Organization. Requirements for adverse reaction reporting. Geneva, Switzerland, 1975.
65. World Health Organization. International drug monitoring: the role of national centres. In *World Health Organization technical report series, no. 498,* Geneva, Switzerland, 1972. Available online: https://apps.who.int/iris/handle/10665/40968 (accessed on 27 July 2022).
66. Lisby, M.; Nielsen, L. P.; Brock, B.; Mainz, J. How should medication errors be defined? Development and test of a definition. *Scand J Public Health* **2012,** *40* (2), 203-10. doi:10.1177/1403494811435489.
67. European Medicines Agency. Clinical safety data management: definitions and standards for expedited reporting ICH topic E2A. 1995. Available online: https://www.ema.europa.eu/en/documents/scientific-guideline/international-conference-harmonisation-technical-requirements-registration-pharmaceuticals-human-use_en-15.pdf (accessed on 27 July 2022).
68. European Medicines Agency. Guideline on good pharmacovigilance practices – module VI. Management and reporting of adverse reactions to medicinal products. Available online: https://www.ema.europa.eu/en/human-regulatory/post-authorisation/pharmacovigilance/medication-errors (accessed on 27 July 2022).
69. Morimoto, T.; Gandhi, T. K.; Seger, A. C.; Hsieh, T. C.; Bates, D. W. Adverse drug events and medication errors: detection and classification methods. *Qual Saf Health Care* **2004,** *13* (4), 306-14. doi:10.1136/qhc.13.4.306.
70. Dequito, A. B.; Mol, P. G.; van Doormaal, J. E.; Zaal, R. J.; van den Bemt, P. M.; Haaijer-Ruskamp, F. M.; Kosterink, J. G. Preventable and non-preventable adverse drug events in hospitalized patients: a prospective chart review in the Netherlands. *Drug Saf* **2011,** *34* (11), 1089-100. doi:10.2165/11592030-000000000-00000.
71. National Coordinating Council for Medication Error Reporting and Prevention (NCC MERP). Available online: http://www.nccmerp.org (accessed on 27 July 2022).
72. Bates, D. W.; Cullen, D. J.; Laird, N.; Petersen, L. A.; Small, S. D.; Servi, D.; Laffel, G.; Sweitzer, B. J.; Shea, B. F.; Hallisey, R.; et al. Incidence of adverse drug events and potential adverse drug events. Implications for prevention. ADE Prevention Study Group. *Jama* **1995,** *274* (1), 29-34.
