# Supplementary material for: Systematic Review of Risk Factors Assessed in Predictive Scoring Tools for Drug-Related Problems in Inpatients
Source: J Clin Med. 2022 Sep 1;11(17):5185. doi: 10.3390/jcm11175185 (PMC9457151; doi:10.3390/jcm11175185)
Supplement: Supplementary file 1 [file jcm-11-05185-s001.zip › Supplementary File S3.pdf]

Supplementary File S3: Inclusion and exclusion criteria

Inclusion criteria	Exclusion criteria
English/German language	Other languages
Original articles / practice reports	Other article types (e.g., reviews, meta-analyses, editorials, summary articles, case reports)
Access to full text	Full text could not be located
Period of time from 01/2011 to 08/2021	Period of time before 01/2011 and after 08/2021
Inpatient setting	Outpatient setting (e.g., ambulatory care, primary health care, nursing homes)
Adult patients	Underaged patients (according to the legal system of the respective country)
Articles investigating medication errors made by physicians	Articles investigating medication errors made by nurses
Predictive scoring tools applied by pharmacists/physicians or by an automatic/electronic assessment	Predictive scoring tools applied by patients themselves (e.g., via a questionnaire)
General predictive scoring tools for identification of patients at risk for drug-related problems which are applicable in the moment of hospitalization/ during hospitalization	Articles only identifying ADE/ADR/ME or even risk factors for drug-related problems but without developing a predictive scoring tool<br>Scoring tools developed for other purposes than identification of patients at risk for DRPs<br><br>Predictive scoring tools focusing only on<br>o A specific demographic patient population (except for geriatric patients)<br>o Patients with specific conditions<br>o A single step in the medication process<br>o Specific DRP
Outcome:<br>o Drug-related problems (including medication errors, adverse drug reactions/events)	Outcome:<br>o Inappropriate prescribing<br>o Underprescribing<br>o Specific DRP (e.g., bleeding due to vitamin K antagonists)<br>o Medication discrepancies<br>o Medication administration errors<br>o General adverse events/ harm not caused by drugs<br>o ADR-related hospital (re-)admissions<br>o DRP at/after discharge from hospital
