# Supplementary material for: Systematic Review of Risk Factors Assessed in Predictive Scoring Tools for Drug-Related Problems in Inpatients
Source: J Clin Med. 2022 Sep 1;11(17):5185. doi: 10.3390/jcm11175185 (PMC9457151; doi:10.3390/jcm11175185)
Supplement: Supplementary file 1 [file jcm-11-05185-s001.zip › Supplementary File S4.pdf]

Supplementary File S4: Detailed information on literature search strategy

Pubmed (Medline):

MeSH Term search combined with free text search, using Boolean operators and the period of time filter 2011-2021: **1934** hits

(("risk assessment"[MeSH:NoExp]) OR ("healthcare failure mode and effect analysis"[MeSH Terms]) OR ("risk adjustment"[MeSH Terms]) OR ("risk assessment"[Title/Abstract]) OR ("risk factors"[MeSH:NoExp]) OR ("risk factor*"[Title/Abstract]) OR ("factor*"[Title/Abstract]) OR ("screening tool*"[Title/Abstract]) OR ("risk score*"[Title/Abstract]) OR ("high risk patient*"[Title/Abstract]) OR ("patients at risk"[Title/Abstract]) OR ("identification of high risk patient*"[Title/Abstract]) OR ("identifying high risk patient*"[Title/Abstract])) AND (("medication errors"[MeSH:NoExp]) OR ("near miss, healthcare"[MeSH Terms]) OR ("drug interactions"[MeSH Terms]) OR ("contraindications, drug"[MeSH Terms]) OR ("drug related side effects and adverse reactions"[MeSH Terms]) OR ("medication error*"[Title/Abstract]) OR ("adverse drug reaction*"[Title/Abstract]) OR ("adverse drug event*"[Title/Abstract]) OR ("drug related problem*"[Title/Abstract]) OR ("medication related problem*"[Title/Abstract]) OR ("drug therapy problem*"[Title/Abstract]) OR ("medication therapy problem*"[Title/Abstract])) AND (("hospitals"[MeSH Terms]) OR ("inpatients"[MeSH Terms]) OR ("hospital*"[Title/Abstract]) OR ("inpatient*"[Title/Abstract]) OR ("hospitalized"[Title/Abstract]) OR ("hospitalised"[Title/Abstract]) OR ("emergency service, hospital"[MeSH Terms]) OR ("emergency department*"[Title/Abstract]) OR ("emergency admission*"[Title/Abstract]) OR ("emergency ward*"[Title/Abstract]) OR ("emergency service*"[Title/Abstract])) NOT (("pediatrics"[MeSH Terms]) OR ("neonatology"[MeSH Terms]) OR ("adolescent"[MeSH Terms]) OR ("child"[MeSH Terms]) OR ("pediatric*"[Title/Abstract]) OR ("paediatric*"[Title/Abstract]) OR ("neonatal"[Title/Abstract]) OR ("neonatology"[Title/Abstract]) OR ("adolescent*"[Title/Abstract]) OR ("child"[Title/Abstract]) OR ("children"[Title/Abstract]) OR ("veterinary medicine"[MeSH Terms]) OR ("veterinary drugs"[MeSH Terms]) OR ("veterinary"[All Fields]) OR ("animal*"[All Fields]) OR ("outpatients"[MeSH Terms]) OR ("ambulatory care"[MeSH Terms]) OR ("ambulatory care facilities"[MeSH Terms]) OR ("outpatient*"[Title/Abstract]) OR ("ambulatory"[Title/Abstract]) OR ("inappropriate prescribing"[MeSH Terms]) OR ("potentially inappropriate medication list"[MeSH Terms]) OR ("inappropriate prescribing"[Title/Abstract]) OR ("potentially inappropriate medication list*"[Title/Abstract]) OR ("inappropriate medication"[Title/Abstract]) OR ("primary health care"[MeSH Terms]) OR ("primary health care"[Title/Abstract]))

Cochrane Library:

Free text search using Boolean operators and the period of time filter 2011-2021: **338** hits

"risk assessment*" AND "drug related problem*"
"risk assessment*" AND "medication related problem*"
"risk assessment*" AND "medication error*"
"risk assessment*" AND "adverse drug reaction*" AND "hospital*"
"risk assessment*" AND "adverse drug event*" AND "hospital*"
"high risk patient*" AND "drug related problem*"
"high risk patient*" AND "medication related problem*"
"high risk patient*" AND "adverse drug reaction*" AND "hospital*"
"high risk patient*" AND "adverse drug event*" AND "hospital*"
"high risk patient*" AND "medication error*" AND "hospital*"

Scopus:

Free text search using Boolean operators and the period of time filter 2011-2021: **47** hits

(risk factor* OR screening tool* OR risk score* OR risk model* OR risk assessment OR risk adjustment OR predictive model* OR prediction score* OR predicition tool* OR risk OR assessment) AND (medication error* OR adverse drug reaction* OR adverse drug event* OR drug related problem* OR medication related problem* OR contraindication* OR drug interaction* OR overdose* OR overdosage* OR overdosing OR overdosing error* OR drug related side effect*) AND (hospital* OR inpatient* OR hospitalised OR hospitalized) AND NOT (pediatric* OR paediatric* OR neonatal OR neonatology OR adolescent* OR child OR children OR veterinary OR animal* OR outpatient* OR ambulatory)
