# Supplementary material for: Systematic Review of Risk Factors Assessed in Predictive Scoring Tools for Drug-Related Problems in Inpatients
Source: J Clin Med. 2022 Sep 1;11(17):5185. doi: 10.3390/jcm11175185 (PMC9457151; doi:10.3390/jcm11175185)
Supplement: Supplementary file 1 [file jcm-11-05185-s001.zip › Supplementary File S5.pdf]

Supplementary File S5: Detailed presentation of all risk factors

			Considered for evaluation as risk factor			
**Drug-related risk factor**	**Citations**	**Assessed in (number of studies)**	**By literature search/expert suggestions**	**By statistical method**	**Tested for inclusion in final scoring tool (e.g. by multivariate analysis due to expert consensus or significance in uni-/bivariate analysis)**	**Risk factor included in final predicitve scoring tool**
**Number of drugs**		**13/14**			**13/13**	**10/13**
Number of medicines precribed (excluding 'when required' and 'once only' medicines, dietary products, non-medicated topical products, wound dressings)	4, 16		✓	✗	✓	✓
Prescribed drugs ≥ 8	6		✗	✓	✓	✗
Number of drugs 0-5	10		✓	✗	✓	✓
Number of drugs 6-11	10		✓	✗	✓	✓
Number of drugs > 12	10		✓	✗	✓	✓
Number of drugs 3-7	10		✓	✗	✓	✗
Number of drugs > 8	10		✓	✗	✓	✗
Number of drugs during hospitalization > 10	14		✗	✓	✓	✓
Number of medications ≥ 8	3, 15		✓	✓	✓	✓
> 5 regular medications	15		✓	**n.a.**	✗	✗
> 10 regular medications	15		✓	**n.a.**	✗	✗
> 15 regular medications	15		✓	**n.a.**	✗	✗
Number of drugs ≤ 5	9		✓	✓	✓	✓
Number of drugs 5-7	9		✓	✓	✓	✓
Number of drugs ≥ 8	9		✓	✓	✓	✓
Number of prescribed drugs	7		✗	✓	✓	✓
Number of medication ≥ 8	12		✗	✓	✓	✓
≥ 6 daily medications	8		✓	✓	✓	✗
0-5 daily medications	8		✓	✓	✗	✗
Number of medications	1		✓	✓	✓	✗
> 8 regular medications (patients with > 8 regular medications, as identified via medication reconciliation process within previous 12 months)	2		✓	✗	✓	✓
Number of medication 0-6	13		✗	✓	✓	✗
Number of medication 7-9	13		✗	✓	✓	✓
Number of medication 10-12	13		✗	✓	✓	✓
Number of medication ≥ 13	13		✗	✓	✓	✓
On ≥ 2 prescription medications	5		✓	✓	✓	✗
On ≥ 3 prescription medications	5		✓	✓	✓	✓
**Drug interactions**		**04/14**			**03/04**	**01/03**
**General**		**02/14**			**01/02**	**00/01**

Drug interactions	4		✓	✗	✗	✗
Drug interactions ≥ 7	6		✗	✓	✓	✗
**Drug interactions with different levels of risk**		**01/14**			**01/01**	**01/01**
Number of drugs with high risk of interaction	10		✓	✗	✓	✓
Number of drugs with low + medium risk of interaction	10		✓	✗	✓	✓
**Drug interactions with different levels of importance/severity**		**02/14**			**02/02**	**00/02**
Risk C drug interactions ≥ 3	6		✗	✓	✓	✗
Risk D drug interactions ≥ 2	6		✗	✓	✓	✗
Risk X drug interactions	6		✗	✓	✗	✗
Serious drug-drug interactions	1		✓	✓	✓	✗
**Drugs with risk of harm**		**02/14**			**01/02**	**01/01**
**General**		**02/14**			**01/02**	**01/01**
Number of drugs with low risk of harm	10		✓	✗	✓	✓
Number of drugs with medium risk of harm	10		✓	✗	✓	✓
Number of drugs with high risk of harm	10		✓	✗	✓	✓
ISMP high-alert medication'/ risk of harm	4		✓	✗	✗	✗
**Specific**		**01/14**			**00/01**	**00/00**
Medicines or combination of medicines that predispose falls	4		✓	✗	✗	✗
Anticholinergic burden	4		✓	✗	✗	✗
**Potentially inappropriate medicines**		**02/14**			**01/02**	**01/01**
Number of potentially inappropriate medicines prescribed	4		✓	✗	✗	✗
1 STOPP medications	8		✓	✓	✓	✓
≥ 2 STOPP medications	8		✓	✓	✓	✓
**Specific drug classes/ drugs**		**11/14**				**11/11**
**ATC A: alimentary tract and metabolism**		**11/14**			**09/11**	**03/09**
Prescription of ≥ 2 ATC A drugs	6		✗	✓	✓	✗
ATC A: alimentary tract and metabolism	14		✗	✓	✓	✗
**Stomatological preparations A01**		**01/14**			**00/01**	**00/00**
Stomatological preparations A01	17		✗	✓	✗	✗
**Drugs for acid related disorders A02**		**04/14**			**02/04**	**00/02**
Gastrointestinal drugs (A02)	1		✓	✓	✓	✗
Drugs for acid related disorders A02	17		✗	✓	✗	✗
**Drugs for peptic ulcer and gastro-oesophageal reflux disease A02B**		**02/14**			**01/02**	**00/01**
Peptic ulcer drugs (prescribed before admission)	11		✗	✓	✗	✗
Peptic ulcer drugs (prescribed after admission)	11		✗	✓	✓	✗
**Proton pump inhibitors A02BC**		**01/14**			**00/01**	**00/00**
PPI	19		✗	✓	✗	✗
**Drugs for functional gastrointestinal disorders A03**		**01/14**			**00/01**	**00/00**
Drugs for functional gastrointestinal disorders A03	17		✗	✓	✗	✗
**Antiemetics and Antinauseants A04**		**01/14**			**00/01**	**00/00**
Antiemetics and Antinauseants A04	17		✗	✓	✗	✗
**Bile and liver therapy A05**		**01/14**			**00/01**	**00/00**
Bile and liver therapy A05	17		✗	✓	✗	✗
**Drugs for constipation A06**		**03/14**			**01/03**	**01/01**

Laxatives (prescribed after admission)	11		✗	✓	✗	✗
Laxatives (prescribed before admission)	11		✗	✓	✓	✓
Laxatives	19		✗	✓	✗	✗
Drugs for constipation A06	17		✗	✓	✗	✗
**Antidiarrheals, intestinal antiinflammatory/ antiifective agents A07**		**04/14**			**00/04**	**00/00**
Antidiarrhoeal medications	15		✓	n.a.	✗	✗
antidiarrheals	19		✗	✓	✗	✗
Antidiarrheals, intestinal antiinflammatory/ antiifective agents A07	17		✗	✓	✗	✗
**Intestinal antiinflammatory agents A07E**						
**Aminosalicylic acid and similar agents**		**01/14**			**n.a.**	**00/01**
Taking ASA or other salicylates (in the last 7 days)	5		✓	✓	n.a.	✗
**Antiobesity preparations, excl. diet products A08**		**01/14**			**00/01**	**00/00**
Antiobesity preparations, excl. diet products A08	17		✗	✓	✗	✗
**Digestives, incl enzymes A09**		**01/14**			**00/01**	**00/00**
Digestives, incl enzymes A09	17		✗	✓	✗	✗
**Drugs used in diabetes A10**		**09/14**			**06/09**	**02/06**
Use of anti-diabetic agents	12		✗	✓	✓	✓
Antidiabetics (prescribed after admission)	11		✗	✓	✗	✗
Antidiabetics (prescribed before admission)	11		✗	✓	✗	✗
Antidiabetic medication	4, 16		✓	✗	✓	✗
Hypoglycemics (A10)	1		✓	✓	✓	✗
Antidiabetic medications (patients receiving 1 or more antidiabetic medications from Pyxis during current admission)	2		✓	✗	✓	✓
antidiabetics	19		✗	✓	✓	✗
Taking insulin/hypoglycemic agents (in the last 7 days)	5		✓	✓	✓	✗
Drugs used in diabetes A10	17		✗	✓	✗	✗
**Insulins and analogues A10A**		**01/14**			**00/01**	**00/00**
Insulin	15		✓	n.a.	✗	✗
**Blood glucose lowering drugs, excl. insulins A10B**		**01/14**			**00/01**	**00/00**
Oral hypoglycaemics	15		✓	n.a.	✗	✗
**Vitamins A11**		**01/14**			**00/01**	**00/00**
Vitamins A11	17		✗	✓	✗	✗
**Mineral supplements A12**		**01/14**			**00/01**	**00/00**
Mineral supplements A12	17		✗	✓	✗	✗
**Tonics A13**		**01/14**			**00/01**	**00/00**
Tonics A13	17		✗	✓	✗	✗
**Anabolic agents for systemic use A14**		**01/14**			**00/01**	**00/00**
Anabolic agents for systemic use A14	17		✗	✓	✗	✗
**Appetite stimulants A15**		**01/14**			**00/01**	**00/00**
Appetite stimulants A15	17		✗	✓	✗	✗
**Other alimentary tract and metabolism products A16**		**01/14**			**00/01**	**00/00**
Other alimentary tract and metabolism products A16	17		✗	✓	✗	✗
**ATC B: blood and hematopoietic organs**		**10/14**			**08/10**	**04/08**
ATC B: blood and hematopoietic organs	14		✗	✓	✓	✗

Prescription of ≥ 3 ATC B drugs	6		✗	✓	✓	✓
**Antithrombotic agents B01(A)**		**08/14**			**06/08**	**03/08**
Antithrombotic agents B01	17		✗	✓	✗	✗
Taking anticoagulant/antiplatelet agent (in the last 7 days)	5		✓	✓	**n.a.**	✗
**Anticoagulants B01AA/B01AB/B01AE/B01AF/B01AX**		**06/14**			**06/06**	**03/06**
Anticoagulants (prescribed before admission)	11		✗	✓	✓	✗
Anticoagulants (prescribed after admission)	11		✗	✓	✗	✗
Anticoagulants	3, 15		✓	✓	✓	✗
Anticoagulants/ direct oral anticoagulants	4, 16		✓	✗	✓	✗
Anticoagulant medications (patients receiving 1 or more anticoagulant medications from Pyxis during current admission)	2		✓	✗	✓	✓
Recent anticoagulant	13		✗	✓	✓	✓
**Vitamin K antagonists B01AA**		**01/14**			**01/01**	**00/01**
Vitamin K antagonists (B01AA)	1		✓	✓	✓	✗
**Heparin group B01AB**		**02/14**			**02/02**	**01/02**
Heparin/LMWH in therapeutic dose (B01AB)	1		✓	✓	✓	✓
**Heparin B01AB01**		**01/14**			**01/01**	**00/01**
Therapeutic heparin	4, 16		✓	✗	✓	✗
**Platelet aggregation inhibitors excl. heparin B01AC**		**03/14**			**02/03**	**00/02**
Antiplatelets	3		✓	✓	✓	✗
Thrombocyte aggregation inhibitors (B01AC)	1		✓	✓	✓	✗
Platelets aggregation inhibitors	19		✗	✓	**n.a.**	**n.a.**
**Antihemorrhagics B02**		**02/14**			**00/02**	**00/00**
Antihemorrhagics B02	17		✗	✓	✗	✗
**Vitamin K and other hemostatics B02B**						
**Vitamin K B02BA**						
**Phytomenadione B02BA01**		**01/14**			**00/01**	**00/00**
Phytomenadione (vitamin K)	15		✓	**n.a.**	✗	✗
**Antianemic preparations B03**		**01/14**			**00/01**	**00/00**
Antianemic preparations B03	17		✗	✓	✗	✗
**Blood substitutes and perfusion solutions B05**		**04/14**			**01/04**	**00/01**
Blood substitutes and perfusion solutions B05	7		✗	✓	✓	✓
**I.v. solutions B05B**						
**Solutions for parenteral nutrition B05BA**		**01/14**			**00/01**	**00/00**
Total parenteral nutrition (TPN)	15		✓	**n.a.**	✗	✗
**I.v. solution additives B05X**						
**Electrolyte solutions B05XA**		**03/14**			**01/03**	**00/01**
Electrolytes or fluids (prescribed before admission)	11		✗	✓	✓	✗
Electrolytes or fluids (prescribed after admission)	11		✗	✓	✓	✗
**Potassium, i.v. B05XA01, B05XA06, B05XA15, B05XA17, B05XA19**		**02/14**			**00/02**	**00/00**
Intravenous potassium	15		✓	**n.a.**	✗	✗
potassium	19		✗	✓	✗	✗
**Other hematological agents B06**		**01/14**			**00/01**	**00/00**
Other hematological agents B06	17		✗	✓	✗	✗

**ATC C: cardiovascular system**		**11/14**				**10/11**		**04/10**
Cardiovascular agents (prescribed after admission)	11		✗	✓	✗		✗	
Cardiovascular agents (prescribed before admission)	11		✗	✓	✗		✗	
Prescription of ≥ 3 ATC C drugs	6		✗	✓	✓		✗	
ATC C: cardiovascular system	14		✗	✓	✓		✓	
Cardiovascular drugs (C)	1		✓	✓	✓		✓	
Cardiovascular medications (patients receiving >3 cardiovascular medications from Pyxis during current admission)	2		✓	✗	✓		✓	
**Cardiac therapy C01**		**07/14**				**04/07**		**01/04**
Cardiac drugs (C01)	1		✓	✓	✓		✗	
Cardiac therapy C01	17		✗	✓	✗		✗	
**Cardiac glycosides C01A**		**01/14**				**01/01**		**00/01**
Cardiac glycosides	12		✗	✓	✓		✗	
**Antiarrhythmics C01B**		**04/14**				**02/04**		**01/02**
Antiarrhythmics	4, 16		✓	✗	✓		✗	
Antiarrhythmics	3		✓	✓	✓		✓	
Taking antiarrhytmics (in the last 7 days)	5		✓	✓	✗		✗	
**Antiarrhythmics class III C01BD**								
**Amiodarone C01BD01**		**01/14**				**00/01**		**00/00**
amiodarone	19		✗	✓	✗		✗	
**Vasodilators used in cardiac diseases C01D**		**01/14**				**01/01**		**00/01**
Antianginals	3		✓	✓	✓		✗	
**Combined antihypertensive agents C02/C03/C07/C08/C09**		**07/14**				**06/07**		**00/06**
Antihypertensives	3		✓	✓	✓		✗	
Antihypertensive agents (prescribed before admission)	11		✗	✓	✓		✗	
Antihypertensive agents (prescribed after admission)	11		✗	✓	✓		✗	
Antihypertensives	12		✗	✓	✓		✗	
Antihypertensive agents (except diuretics)	19		✗	✓	✓		✗	
Taking antihypertensive/diuretics (in the last 7 days)	5		✓	✓	✓		✗	
**Antihypertensives C02**		**01/14**				**00/01**		**00/00**
Antihypertensives C02	17		✗	✓	✗		✗	
**Diuretics C03**		**05/14**				**02/05**		**00/02**
Diuretics	3		✓	✓	✓		✗	
Diuretics (prescribed before admission)	11		✗	✓	✗		✗	
Diuretics (prescribed after admission)	11		✗	✓	✗		✗	
Diuretics (C03)	1		✓	✓	✓		✗	
diuretics	19		✗	✓	✗		✗	
Diuretics C03	17		✗	✓	✗		✗	
**Beta blocking agents C07**		**03/14**				**02/03**		**00/02**
Betablockers	12		✗	✓	✓		✗	
Betablockers (C07)	1		✓	✓	✓		✗	
Beta blocking agents C07	17		✗	✓	✗		✗	
**Calcium channel blockers C08**		**01/14**				**00/01**		**00/00**
Calcium channel blockers C09	17		✗	✓	✗		✗	

**Agents acting on the renin-angiotensin system C09**		**03/14**			**02/03**	**00/02**
Angiotensine converting enzymes inhibitors	12		✖	✓	✓	✖
RAS inhibitors (C09)	1		✓	✓	✓	✖
Agents acting on the renin-angiotensin system C09	17		✖	✓	✖	✖
**Peripheral vasodilators C04**		**01/14**			**00/01**	**00/00**
Peripheral vasodilators C04	17		✖	✓	✖	✖
**Vasoprotectives C05**		**01/14**			**00/01**	**00/00**
Vasoprotectives C05	17		✖	✓	✖	✖
**Other cardiovascular agents C06**		**01/14**			**00/01**	**00/00**
Other cardiovascular agents C06	17		✖	✓	✖	✖
**Lipid modifying agents C10**		**05/14**			**03/05**	**00/03**
Cholesterol lowering medications	3		✓	✓	✓	✖
Dyslipidemic agents (prescribed before admission)	11		✖	✓	✓	✖
Dyslipidemic agents (prescribed after admission)	11		✖	✓	✖	✖
Antilipaemicae (C10)	1		✓	✓	✓	✖
Cholesterol lowering medications (except statins & fibrates)	19		✖	✓	✖	✖
Lipid modifying agents C10	17		✖	✓	✖	✖
**Lipid modifying agents, plain C10A**		**01/14**			**00/01**	**00/00**
**HMG CoA reductase inhibitors C10AA**		**01/14**			**00/01**	**00/00**
statins	19		✖	✓	✖	✖
**Fibrates C10AB**		**01/14**			**00/01**	**00/00**
fibrates	19		✖	✓	✖	✖
**ATC D: dermatological therapy**		**03/14**			**01/03**	**00/03**
ATC D: dermatological therapy	14		✖	✓	✓	✖
Prescription of ATC D drugs	6		✖	✓	✖	✖
**Antifungals for dermatological use D01**		**01/14**			**00/01**	**00/01**
Antifungals for dermatological use D01	17		✖	✓	✖	✖
**Emollients and protectives D02**		**01/14**			**00/01**	**00/01**
Emollients and protectives D02	17		✖	✓	✖	✖
**Preparation for treatment of wounds and ulcers D03**		**01/14**			**00/01**	**00/01**
Preparation for treatment of wounds and ulcers D03	17		✖	✓	✖	✖
**Antipruritics, incl. antihistamines, anesthetics, etc. D04**		**01/14**			**00/01**	**00/01**
Antipruritics, incl. antihistamines, anesthetics, etc. D04	17		✖	✓	✖	✖
**Antipsoriatics D05**		**01/14**			**00/01**	**00/01**
Antipsoriatics D05	17		✖	✓	✖	✖
**Antibiotics and chemotherapeutics for dermatological use D06**		**01/14**			**00/01**	**00/01**
Antibiotics and chemotherapeutics for dermatological use D06	17		✖	✓	✖	✖
**Corticosteroids, dermatological preparations D07**		**01/14**			**00/01**	**00/01**
Corticosteroids, dermatological preparations D07	17		✖	✓	✖	✖
**Antiseptics and disinfectants D08**		**01/14**			**00/01**	**00/01**
Antiseptics and disinfectants D08	17		✖	✓	✖	✖
**Medicated dressings D09**		**01/14**			**00/01**	**00/01**
Medicated dressings D09	17		✖	✓	✖	✖
**Anti-acne preparations D10**		**01/14**			**00/01**	**00/01**

Anti-acne preparations D10	17		✖	✓	✖	✖
**Other dermatological preparations D11**		**01/14**			**00/01**	**00/01**
Other dermatological preparations D11	17		✖	✓	✖	✖
**ATC G: genito urinary system and sex hormones**		**03/14**			**01/03**	**00/03**
ATC G: genitourinary therapy (including hormone therapy)	14		✖	✓	✓	✖
Prescription of ATC G drugs	6		✖	✓	✖	✖
**Gynecological antiinfectives and antiseptics G01**		**01/14**			**00/01**	**00/01**
Gynecological antiinfectives and antiseptics G01	17		✖	✓	✖	✖
**Other gynecologicals G02**		**01/14**			**00/01**	**00/01**
Other gynecologicals G02	17		✖	✓	✖	✖
**Sex hormones and modulators of the genital system G03**		**01/14**			**00/01**	**00/01**
Sex hormones and modulators of the genital system G03	17		✖	✓	✖	✖
**Urologicals G04**		**01/14**			**00/01**	**00/01**
Urologicals G04	17		✖	✓	✖	✖
**ATC H: systemic hormonal preparations, excl. sex hormones and insulins**		**06/14**			**02/06**	**01/02**
Prescription of ≥ 2 ATC H drugs (systemic hormonal preparations, excluding sex hormones and insulins)	6		✖	✓	✓	✖
ATC H: hormone therapy	14		✖	✓	✓	✓
**Pituitary and hypothalamic hormones and analogues H01**		**01/14**			**00/01**	**00/00**
Pituitary and hypothalamic hormones and analogues H01	17		✖	✓	✖	✖
**Corticosteroids for systemic use H02**		**04/14**			**00/04**	**00/00**
Corticosteroids (H02)	1		✓	✓	✖	✖
Corticosteroids	15		✓	**n.a.**	✖	✖
corticosteroids	19		✖	✓	✖	✖
Corticosteroids for systemic use H02	17		✖	✓	✖	✖
**Thyroid therapy H03**		**01/14**			**00/01**	**00/00**
Thyroid therapy H03	17		✖	✓	✖	✖
**Pancreatic hormones H04**		**01/14**			**00/01**	**00/00**
Pancreatic hormones H04	17		✖	✓	✖	✖
**Calcium homeostasis H05**		**01/14**			**00/01**	**00/00**
Calcium homeostasis H05	17		✖	✓	✖	✖
**ATC J: systemic, anti-infectious therapy**		**10/14**			**07/10**	**03/07**
Other systemic antimicrobials (excluding aminoglycosides and glycopeptides)	4, 16		✓	✖	✓	✓
Antimicrobials	3, 15		✓	✓	✓	✖
ATC J: systemic, anti-infectious therapy	14		✖	✓	✓	✓
Prescription of ATC J drugs	6		✖	✓	✖	✖
Antiinfective medicines	12		✖	✓	✓	✖
**Antibacterials for systemic use J01 and Antimycotics for systemic use J02**		**06/14**			**04/06**	**02/04**
Antimicrobials (J01, J02)	1		✓	✓	✓	✖
**Antibacterials for systemic use J01**		**05/14**			**03/05**	**02/03**
Antibiotics (prescribed before admission)	11		✖	✓	✓	✖
Antibiotics (prescribed after admission)	11		✖	✓	✖	✖
Having recieved antibiotics in last 3 months	4		✓	✖	✖	✖
antibiotics	19		✖	✓	✖	✖

On antibiotics in the last 7 days	5		✓	✓	✓	✓
On antibiotics: 1-4 weeks ago	5		✓	✓	✗	✗
On antibiotics: 1-3 months ago	5		✓	✓	✗	✗
On antibiotics: >3 months ago	5		✓	✓	✗	✗
Antibacterials for systemic use J01	17		✗	✓	✗	✗
**Aminoglycoside antibacterials J01G and Glycopeptide antibacterials J01XA**		**01/14**			**01/01**	**01/01**
Aminoglycosides and glycopeptides	4, 16		✓	✗	✓	✓
**Antimycotics for systemic use J02**		**01/14**			**00/01**	**00/00**
Antimycotics for systemic use J02	17		✗	✓	✗	✗
**Antimycobacterials J04**		**01/14**			**00/01**	**00/00**
Antimycobacterials J04	17		✗	✓	✗	✗
**Antivirals for systemic use J05**		**01/14**			**00/01**	**00/00**
Antivirals for systemic use J05	17		✗	✓	✗	✗
**Immune sera and immunoglobulins J06**		**01/14**			**00/01**	**00/00**
Immune sera and immunoglobulins J06	17		✗	✓	✗	✗
**Vaccines J07**		**01/14**			**00/01**	**00/00**
Vaccines J07	17		✗	✓	✗	✗
**ATC L: antineoplastic therapy and immuno-modulatory agents**		**09/14**			**04/09**	**01/04**
Prescription of ATC L drugs	6		✗	✓	✗	✗
ATC L: antineoplastic therapy and immuno-modulatory agents	14		✗	✓	✓	✗
**Antineoplastic agents L01**		**06/14**			**01/06**	**00/01**
Cytotoxics	4, 16		✓	✗	✓	✗
Chemotherapy (L01)	1		✓	✓	✗	✗
Cytotoxics	15		✓	**n.a.**	✗	✗
chemotherapy	19		✗	✓	✗	✗
Taking chemotherapeutic agents (in the last 7 days)	5		✓	✓	✗	✗
Antineoplastic agents L01	17		✗	✓	✗	✗
**Endocrine therapy L02**		**01/14**			**00/01**	**00/00**
Endocrine therapy L02	17		✗	✓	✗	✗
**Immunostimulants L03**		**01/14**			**00/01**	**00/00**
Immunostimulants L03	17		✗	✓	✗	✗
**Immunosuppressants L04(A)**		**04/14**			**03/04**	**01/03**
Immunosuppression therapy	11		✗	✓	✓	✗
Immunosuppressants	3, 15		✓	✓	✓	✓
Immunosuppressants (excluding corticosteroids)	4, 16		✓	✗	✓	✗
Immunosuppressants L04	17		✗	✓	✗	✗
**ATC M: musculo-skeletal system**		**08/14**			**02/08**	**00/02**
Prescription of ATC M drugs	6		✗	✓	✗	✗
ATC M: musculoskeletal system	14		✗	✓	✓	✗
**Antiinflammatory and antirheumatic products M01**		**06/14**			**01/06**	**00/01**
anti-inflammatory drugs	19		✗	✓	**n.a.**	✗
Antiinflammatory and antirheumatic products M01	17		✗	✓	✗	✗
**NSAIDs M01A**		**04/14**			**01/04**	**00/01**
NSAIDs (prescribed before admission)	11		✗	✓	✗	✗

NSAIDs (prescribed after admission)	11		✗	✓	✓		✗	
NSAIDs (M01A)	1		✓	✓	✗		✗	
Non-steroidal anti-inflammatory drugs (NSAIDs)	15		✓	n.a.	✗		✗	
Taking NSAID (in the last 7 days)	5		✓	✓	n.a.		✗	
**Topical products for joint and muscular pain M02**		**01/14**				**00/01**		**00/00**
Topical products for joint and muscular pain M02	17		✗	✓	✗		✗	
**Muscle relaxants M03**		**01/14**				**00/01**		**00/00**
Muscle relaxants M03	17		✗	✓	✗		✗	
**Antigout preparations M04**		**01/14**				**00/01**		**00/00**
Antigout preparations M04	17		✗	✓	✗		✗	
**Drugs for treatment of bone diseases M05**		**01/14**				**00/01**		**00/00**
Drugs for treatment of bone diseases M05	17		✗	✓	✗		✗	
**Other drugs for disorders of the muskulo-skeletal system M09**		**01/14**				**00/01**		**00/00**
Other drugs for disorders of the muskulo-skeletal system M09	17		✗	✓	✗		✗	
**ATC N: nervous system**		**11/14**				**10/11**		**06/10**
Prescription of ATC N drugs	6		✗	✓	✗		✗	
ATC N: nervous system	14		✗	✓	✓		✗	
**Anesthetics N01**		**01/14**				**00/01**		**00/00**
Anesthetics N01	17		✗	✓	✗		✗	
**Analgesics N02**		**08/14**				**07/08**		**02/07**
Analgesics (prescribed before admission)	11		✗	✓	✓		✗	
Analgesics (prescribed after admission)	11		✗	✓	✗		✗	
Analgesics N02	17		✗	✓	✗		✗	
**Opioids N02A**		**06/14**				**06/06**		**02/06**
Opioids	3, 15		✓	✓	✓		✗	
Opiates (excluding codeine, tramadol and dihydrocodeine)	4, 16		✓	✗	✓		✗	
Opioid analgesics	12		✗	✓	✓		✗	
Opioids (N02A)	1		✓	✓	✓		✓	
opioids	13		✗	✓	✓		✗	
Opioid medications (patients receiving >1 opioid medication from Pyxis during current admission)	2		✓	✗	✓		✓	
**Other analgesics and antipyretics N02B**								
**Anilides N02BE**								
**Paracetamol N02BE01**		**01/14**				**00/01**		**00/00**
paracetamol	19		✗	✓	✗		✗	
**Antiepileptics N03(A)**		**07/14**				**02/07**		**02/02**
Epilepsy medicines	4, 16		✓	✗	✓		✓	
Antiepileptics (N03)	1		✓	✓	✗		✗	
Antiepileptic medications (patients receiving 1 or more antiepileptic medications from Pyxis during current admission)	2		✓	✗	✓		✓	
Anticonvulsants	15		✓	n.a.	✗		✗	
anticonvulsants	19		✗	✓	✗		✗	
Taking seizure medications (in the last 7 days)	5		✓	✓	✗		✗	
Antiepileptics N03	17		✗	✓	✗		✗	

**Anti-parkinson drugs N04**		**03/14**			**00/03**	**00/0**
Parkinson´s medications	15		✓	n.a.	✗	✗
Parkinson's disease therapy // Anti-Parkinson drugs	19		✗	✓	✗	✗
Anti-parkinson drugs N04	17		✗	✓	✗	✗
**Psycholeptics and Psychoanaleptics N05/N06**		**07/14**			**07/07**	**03/07**
CNS agents (N05/N06)	1		✓	✓	✓	✗
**Psycholeptics N05**		**06/14**			**06/06**	**03/06**
Psycholeptics N05	7		✗	✓	✓	✓
**Antipsychotics N05A**		**05/14**			**05/05**	**02/05**
Antipsychotics (prescribed before admission)	11		✗	✓	✗	✗
Antipsychotics (prescribed after admission)	11		✗	✓	✓	✗
Antipsychotics	3, 15		✓	✓	✓	✓
Antipsychotics	4, 16		✓	✗	✓	✗
Antipsychotics (N05A)	1		✓	✓	✓	✗
Antipsychotics	19		✗	✓	✓	✓
**Other antipsychotics N05AX**		**01/14**			**00/01**	**00/00**
atypical neuroleptics	19		✗	✓	✗	✗
**Lithium N05AN**		**01/14**			**01/01**	**00/01**
Lithium	4, 16		✓	✗	✓	✗
**Anxiolytics N05B**		**03/14**			**00/03**	**00/00**
Anxiolytics (N05B)	1		✓	✓	✗	✗
anxylotics	19		✗	✓	✗	✗
**Benzodiazepine derivates N05BA**		**01/14**			**00/01**	**00/00**
Benzodiazepines	15		✓	n.a.	✗	✗
**Carbamates N05BC**						
**Meprobamate N05BC01**		**01/14**			**00/01**	**00/00**
meprobamate	19		✗	✓	✗	✗
**Hypnotics and Sedatives N05C**		**02/14**			**02/02**	**00/02**
Sedatives (prescribed before admission)	11		✗	✓	✓	✗
Sedatives (prescribed after admission)	11		✗	✓	✗	✗
hypnotics and sedatives	19		✗	✓	✓	✗
**Psychoanaleptics N06**		**05/14**			**02/05**	**00/02**
Psychoanaleptics N06	17		✗	✓	✗	✗
**Antidepressants N06A**		**04/14**			**02/04**	**00/02**
Antidepressants	4, 16		✓	✗	✓	✗
Antidepressants (N06A)	1		✓	✓	✗	✗
Antidepressants	15		✓	n.a.	✗	✗
antidepressants	13		✗	✓	✓	✗
**Anti-dementia drugs N06D**		**01/14**			**00/01**	**00/00**
Alzheimer's disease treatment // Anti-dementia drugs	19		✗	✓	✗	✗
**Other nervous system drugs N07**		**01/14**			**00/01**	**00/00**
Other nervous system drugs N07	17		✗	✓	✗	✗
**Other ATC N**		**01/14**			**00/01**	**00/00**
Taking opioids or benzodiazepines (in the last 7 days)	5		✓	✓	✓	✗

**ATC P: antiparasitic products, insecticides, and repellants**		**03/14**				**00/03**		**00/00**
Prescription of ATC P drugs	6		✗	✓	✗		✗	
ATC P: antiparasitic products, insecticides, and repellants	14		✗	✓	✗		✗	
**Antiprotozoals P01**		**01/14**				**00/01**		**00/00**
Antiprotozoals P01	17		✗	✓	✗		✗	
**Antihelmintics P02**		**01/14**				**00/01**		**00/00**
Antihelmintics P02	17		✗	✓	✗		✗	
**Ectoparasiticides, incl. scabicides, insecticides and repellents P03**		**01/14**				**00/01**		**00/00**
Ectoparasiticides, incl. scabicides, insecticides and repellents P03	17		✗	✓	✗		✗	
**ATC R: respiratory system**		**05/14**				**03/05**		**01/03**
ATC R: respiratory system	14		✗	✓	✓		✗	
Prescription of ATC R drugs	6		✗	✓	✓		✓	
**Nasal preparations R01**		**01/14**				**00/01**		**00/00**
Nasal preparations R01	17		✗	✓	✗		✗	
**Throat preparations R02**		**01/14**				**00/01**		**00/00**
Throat preparations R02	17		✗	✓	✗		✗	
**Drugs for obstructive airway diseases R03**		**02/14**				**01/02**		**00/01**
Drugs for obstructive airway diseases R03	17		✗	✓	✗		✗	
**Adrenergics, inhalants R03A**								
**Other systemic drugs for obstructive airway diseases R03D**								
**Xanthines R03DA**								
**Theophylline R03DA04 and Aminophylline R03DA05**		**01/14**				**01/01**		**00/01**
Theophylline and aminophylline	4, 16		✓	✗	✓		✗	
**Chest rubs and other inhalants R04**		**01/14**				**00/01**		**00/00**
Chest rubs and other inhalants R04	17		✗	✓	✗		✗	
**Cough and cold preparations R05**		**01/14**				**00/01**		**00/00**
Cough and cold preparations R05	17		✗	✓	✗		✗	
**Antihistamines for systemic use R06(A)**		**02/14**				**00/02**		**00/00**
Antihistamines	15		✓	**n.a.**	✗		✗	
Antihistamines for systemic use R06	17		✗	✓	✗		✗	
**Other respiratory system products R07**		**01/14**				**00/01**		**00/00**
Other respiratory system products R07	17		✗	✓	✗		✗	
**ATC S: sensory organs**		**03/14**				**01/03**		**01/01**
Prescription of ATC S drugs	6		✗	✓	✗		✗	
ATC S: sensory organs	14		✗	✓	✓		✓	
**Ophthalmologicals S01**		**01/14**				**00/01**		**00/00**
Ophthalmologicals S01	17		✗	✓	✗		✗	
**Otologicals S02**		**01/14**				**00/01**		**00/00**
Otologicals S02	17		✗	✓	✗		✗	
**Ophthalmological and otological preparations S03**		**01/14**				**00/01**		**00/00**
Ophthalmological and otological preparations S03	17		✗	✓	✗		✗	
**ATC V: various**		**05/14**				**01/05**		**01/01**
Prescription of ATC V drugs	6		✗	✓	✗		✗	
ATC V: various	14		✗	✓	✓		✓	

**Allergens V01**		**01/14**				**00/01**	**00/00**
Allergens V01	17		✘	✓	✘		✘
**All other therapeutic products V03(A)**		**03/14**				**00/03**	**00/00**
All other therapeutic products V03	17		✘	✓	✘		✘
**Antidotes V03AB**		**02/14**				**00/02**	**00/00**
Use of an antidote e.g. naloxone, vitamin K	4		✓	✘	✘		✘
**Protamine V03AB14**		**01/14**				**00/01**	**00/00**
Protamine Sulphate	15		✓	**n.a.**	✘		✘
**Naloxone V03AB15**		**01/14**				**00/01**	**00/00**
Naloxone	15		✓	**n.a.**	✘		✘
**Acetylcysteine V03AB23**		**01/14**				**00/01**	**00/00**
Acetylcysteine	15		✓	**n.a.**	✘		✘
**Digitalis antitoxin V03AB24**		**01/14**				**00/01**	**00/00**
Digibind	15		✓	**n.a.**	✘		✘
**Flumazenil V03AB25**		**01/14**				**00/01**	**00/00**
Flumazenil	15		✓	**n.a.**	✘		✘
**Idarucizumab V03AB37**		**01/14**				**00/01**	**00/00**
Idarucizumab (Praxbind)	15		✓	**n.a.**	✘		✘
**Iron chelating agents V03AC**							
**Deferoxamine V03AC01**		**01/14**				**00/01**	**00/00**
Desferrioxamine (Desferal)	15		✓	**n.a.**	✘		✘
**Detoxifying agents for antineoplastic treatment V03AF**							
**Folinic acid/ Folinic acid salts V03AF03, V03AF04, V03Af06, V03AF10**		**01/14**				**00/01**	**00/00**
Folinic acid	15		✓	**n.a.**	✘		✘
**Diagnostic agents V04**		**01/14**				**00/01**	**00/00**
Diagnostic agents V04	17		✘	✓	✘		✘
**General nutrients V06**		**01/14**				**00/01**	**00/00**
General nutrients V06	17		✘	✓	✘		✘
**All other non-therapeutic products V07**		**01/14**				**00/01**	**00/00**
All other non-therapeutic products V07	17		✘	✓	✘		✘
**Contrast media V08**		**01/14**				**00/01**	**00/00**
Contrast media V08	17		✘	✓	✘		✘
**Diagnostic radiopharmaceuticals V09**		**01/14**				**00/01**	**00/00**
Diagnostic radiopharmaceuticals V09	17		✘	✓	✘		✘
**Therapeutic radiopharmaceuticals V10**		**01/14**				**00/01**	**00/00**
Therapeutic radiopharmaceuticals V10	17		✘	✓	✘		✘
**Surgical dressings V20**		**01/14**				**00/01**	**00/00**
Surgical dressings V20	17		✘	✓	✘		✘
**Homeopathics and anthroposophics V60**		**01/14**				**00/01**	**00/00**
Homeopathics and anthroposophics V60	17		✘	✓	✘		✘
**Formulations V70**		**01/14**				**00/01**	**00/00**
Formulations V70	17		✘	✓	✘		✘
**Special groups V90**		**01/14**				**00/01**	**00/00**
Special groups V90	17		✘	✓	✘		✘

**OTC/ herbal medicine**		**02/14**				**00/02**		**00/00**
OTC/ herbal medicine use	4		✓	✗	✗		✗	
Use of herbal remedies	5		✓	✓	**n.a.**		✗	
Use of OTC supplements	5		✓	✓	✗		✗	
**Other**		**01/14**				**01/01**		**00/01**
Other (Clozapine, antiretrovirals, medicines for Parkinson´s disease)	4, 16		✓	✗	✓		✗	
**Route of application**		**02/14**				**02/02**		**01/02**
**Parenteral application**		**02/14**				**02/02**		**01/02**
Parenteral administration route	4, 16		✓	✗	✓		✗	
**Intravenous application**		**01/14**				**01/01**		**01/01**
Prescription of intreavenous drugs	6		✗	✓	✓		✓	
**Subcutaneous application**		**01/14**				**01/01**		**00/01**
Prescriptions of subcutaneous drugs ≥ 2	6		✗	✓	✓		✗	
**Other application**		**01/14**				**01/01**		**01/01**
Prescription of ≥ 6 oral drugs	6		✗	✓	✓		✓	
Prescription of inhaled drugs	6		✗	✓	✗		✗	
Prescription of ocular drugs	6		✗	✓	✗		✗	
Prescription of enteral tube drugs	6		✗	✓	✗		✗	
Prescription of sublingual drugs	6		✗	✓	✗		✗	
Prescription of nasal drugs	6		✗	✓	✗		✗	
Prescription of rectal drugs	6		✗	✓	✗		✗	
Prescription of topical drugs	6		✗	✓	✗		✗	
**Previous ADR**		**03/14**				**03/03**		**02/03**
Prior history of ADR	6		✗	✓	✓		✓	
Previous ADR	9		✓	✓	✓		✓	
History of previous ADR	12		✓	✓	✓		✗	
**Previous allergy**		**04/14**				**02/04**		**02/02**
Previous allergy	3, 15		✓	✓	✓		✓	
History of allergy (present)	11		✗	✓	✗		✗	
Previous allergy	4, 16		✓	✗	✓		✓	
Previous drug allergies	12		✗	✓	✗		✗	
**Narrow therapeutic index**		**04/14**				**02/04**		**01/02**
Drugs with a narrow therapeutic index	12		✓	✗	✓		✗	
Narrow therapeutic index' medicines	4		✓	✗	✗		✗	
TDM medications (patients receiving 1 or more medications requiring TDM from Pyxis during current admission)	2		✓	✗	✓		✓	
**Specific drugs**		**01/14**				**00/01**		**00/00**
Phenytoin	15		✓	**n.a.**	✗		✗	
Lithium	15		✓	**n.a.**	✗		✗	
Clozapine	15		✓	**n.a.**	✗		✗	
Tacrolimus	15		✓	**n.a.**	✗		✗	
Cyclosporin	15		✓	**n.a.**	✗		✗	
Vancomycin	15		✓	**n.a.**	✗		✗	
Tobramycin	15		✓	**n.a.**	✗		✗	

Gentamicin	15	✓	**n.a.**	✘	✘
Amikacin	15	✓	**n.a.**	✘	✘
Theophylline	15	✓	**n.a.**	✘	✘
Digoxin	15	✓	**n.a.**	✘	✘
**Others (named <2x)**					
Drug-drug incompatibility	6	✘	✓	✘	✘
Medication Regimen Complexity Index	4	✓	✘	✘	✘
Medicine use 'off label'	4	✓	✘	✘	✘
Length and appropriateness of antibiotic treatment	4	✓	✘	✘	✘
Constituents in formulations that may be pharmacologically active	4	✓	✘	✘	✘
Requirement to manipulate the medicine before administration	4	✓	✘	✘	✘
Outdated Pyxis profile (patients whose Pyxis medication profiles have not been updated during past 72 hours)	2	✓	✘	✓	✓
Prescribing for Elderly (patients with age of >80 years and admission source for current encounter designated in PIMS as “rest home” or “private hospital” or specified other hospitals and taking >8 regular medications on admission and received specified high-risk medications from Pyxis in past 30 days)	2	✓	✘	✓	✓
Drug dose (high versus low)	4	✓	✘	✘	✘
Dosing frequency of medication	4	✓	✘	✘	✘
Irregular dose and administration	4	✓	✘	✘	✘
Over sedation	15	✓	**n.a.**	✘	✘
Clinical trial medication	15	✓	**n.a.**	✘	✘

			Considered for evaluation as risk factor			
**Diagnosis-related risk factor**	**Citations**	**Assessed in (number of studies)**	**By literature search/expert suggestions**	**By statistical method**	**Tested for inclusion in final scoring tool (e.g. by multivariate analysis due to expert consensus or significance in uni-/bivariate analysis)**	**Risk factor included in final predicitve scoring tool**
**Comorbidities**		**10/14**			**09/10**	**05/09**
**Number of comorbidities**		**08/14**			**07/08**	**04/07**
Number of comorbidities	4, 16		✓	×	✓	✓
≥ 4 Comorbid conditions	9		✓	✓	✓	✓
Number of comorbidities ≥ 4	12		✓	✓	✓	×
≥ 4 Comorbid conditions	8		✓	✓	✓	✓
> 1 comorbid conditions	15		✓	n.a.	×	×
> 2 comorbid conditions	15		✓	n.a.	×	×
> 3 comorbid conditions	15		✓	n.a.	×	×
> 4 comorbid conditions	15		✓	n.a.	×	×
number of comorbid conditions	13, 18		×	✓	✓	×
≥ 1 Comorbid conditions	5		✓	✓	✓	✓
Comorbidity (yes)	10		✓	×	✓	×
**Charlson Comorbidity Index**		**04/14**			**03/04**	**01/03**
Charlson index = 1	14		×	✓	✓	×
Charlson index = 2	14		×	✓	✓	✓
Comorbidity (Charlson Comorbidity Index)	10		✓	×	✓	×
Charlson´s index ≥ 4	6		×	✓	✓	×
Charlson survival probability	6		×	✓	×	×
Comorbidity index	4		✓	×	×	×
**DRG weight**		**02/14**			**01/02**	**00/01**
DRG weight	14		×	✓	✓	×
DRG weight	4		✓	×	×	×
**Other ways of assessing comorbidities**		**01/14**			**01/01**	**00/01**
Higher levels on the modifed cumulative illness rating scale (mCIRS)	8		✓	✓	✓	×
**Specific diseases**		**11/14**				
**Diseases and disorders of the nervous system**		**11/14**			**08/11**	**04/08**
Nervous system and mental disorders (as primary diagnosis)	4		✓	×	✓	✓
**Nervous system Disease**		**05/14**			**02/05**	**02/02**
MDC nervous system	14		×	✓	✓	✓
Neurological disorder (e.g. Parkinson´s disease)	15		✓	n.a.	×	×
chronic disease: neurological	18		×	✓	×	×
**Hemiplegia**		**02/14**			**01/02**	**01/01**
Hemiplegia (Charlson comorbidity index)	6		×	✓	×	×
Hemiplegia (Charlson comorbidity index)	11		×	✓	✓	✓

**Mental disorders and diseases**		**10/14**				**07/10**		**02/07**
Main diagnosis classified in Chapter V ICD 10 - Mental and behavioral disorders	6		✗	✓	✓		✗	
Mental health history (patients with mental health history within previous 12 months (Note: exclusion criteria for inpatients under care of mental health services))	2		✓	✗	✓		✓	
Mental health history	15		✓	n.a.	✗		✗	
History of psychiatric problems	5		✓	✓	✓		✗	
Presence of mental health issues	5		✓	✓	✓		✗	
**Dementia/Alzheimer**		**08/14**				**03/08**		**01/03**
Dementia	9		✓	✓	✗		✗	
Dementia (Charlson comorbidity index)	11		✗	✓	✓		✓	
History of dementia	4, 16		✓	✗	✓		✗	
Dementia (Charlson comorbidity index)	6		✗	✓	✗		✗	
Dementia	8		✓	✓	✓		✗	
Dementia	15		✓	n.a.	✗		✗	
chronic disease: dementia	18		✗	✓	✗		✗	
Dementia (other than Alzheimer)	12		✗	✓	✗		✗	
Alzheimer	12		✗	✓	✗		✗	
**Depression**		**02/14**				**01/02**		**00/01**
Depression	9		✓	✓	✓		✗	
Depression	12		✗	✓	✗		✗	
**Opthalmological diseases**		**01/14**				**00/01**		**00/00**
chronic disease: ophtalmological	18		✗	✓	✗		✗	
**Diseases and disorders of the blood and blood forming organs and immunological disorders**		**02/14**				**01/02**		**00/01**
**Diseases of the blood and blood forming organs**		**01/14**				**00/01**		**00/00**
Anaemia	12		✗	✓	✗		✗	
**Immunological disorders**		**01/14**				**01/01**		**00/01**
Rheumatologic disease (Charlson comorbidity index)	11		✗	✓	✓		✗	
**Diseases and disorders of the circulatory system**		**11/14**				**11/11**		**04/11**
MDC circulatory system	14		✗	✓	✓		✓	
Main diagnosis classified in Chapter IX ICD 10 - Diseases of the circulatory system	6		✗	✓	✓		✗	
**Cardiovascular Disease**		**10/14**				**09/10**		**02/09**
Cardiovascular system (as primary diagnosis)	4		✓	✗	✓		✗	
Cardiovascular disease (Charlson comorbidity index)	6		✗	✓	✗		✗	
chronic disease: cardiovascular	13, 18		✗	✓	✓		✗	
**Myocardial infarction**		**03/14**				**00/03**		**00/00**
Myocardial infarction (Charlson comorbidity index)	11		✗	✓	✗		✗	
Myocardial infarction (Charlson comorbidity index)	6		✗	✓	✗		✗	
Recent STEMI/NSTEMI	15		✓	n.a.	✗		✗	
**Heart failure**		**08/14**				**06/08**		**02/06**
Heart failure (Charlson comorbidity index)	11		✗	✓	✓		✗	
Heart failure	9		✓	✓	✓		✓	
Congestive cardiac failure	12		✓	✓	✓		✗	
Chronic heart failure (Charlson comorbidity index)	6		✗	✓	✗		✗	

Heart failure	8		✓	✓	✓	✗
CCM Congestive heart failure (patients actively enrolled or previously enrolled in CHF CCM program within past 12 months)	2		✓	✗	✓	✓
Congestive heart failure	15		✓	n.a.	✗	✗
History of heart failure	5		✓	✓	✓	✗
**Atrial fibrillation**		**03/14**			**00/03**	**00/00**
Atrial fibrillation	15		✓	n.a.	✗	✗
History of atrial fibrillation	5		✓	✓	n.a.	✗
Atrial fibrillation	12		✗	✓	✗	✗
**Ischaemic heart disease**		**02/14**			**01/02**	**00/01**
Ischaemic heart disease	3, 15		✓	✓	✓	✗
Ischaemic heart disease	12		✗	✓	✗	✗
**Hypertension**		**02/14**			**00/02**	**00/00**
Hypertension	15		✓	n.a.	✗	✗
Hypertension	12		✗	✓	✗	✗
**Hypotension**		**01/14**			**00/01**	**00/00**
Hypotension	15		✓	n.a.	✗	✗
**Peripheral vascular diseases**		**02/14**			**00/02**	**00/00**
Peripheral vascular disease (Charlson comorbidity index)	11		✗	✓	✗	✗
Peripheral vascular disease (Charlson comorbidity index)	6		✗	✓	✗	✗
**Cerebrovascular diseases**		**04/14**			**01/04**	**01/01**
Cerebrovascular disease (Charlson comorbidity index)	11		✗	✓	✗	✗
CCM Cerebrovascular disease (patients actively enrolled or previously enrolled in CVD CCM program within past 12 months)	2		✓	✗	✓	✓
**Stroke/TIA**		**02/14**			**00/02**	**00/00**
Stroke	15		✓	n.a.	✗	✗
Transient Ischaemic Attack (TIA)	15		✓	n.a.	✗	✗
Previous stroke	12		✗	✓	✗	✗
Previous TIA	12		✗	✓	✗	✗
**Other thromboembolisms**		**02/14**			**02/02**	**01/02**
Venous or arterial thrombosis and/or embolism	3, 15		✓	✓	✓	✓
recent VTE	13		✗	✓	✓	✗
**Diseases and disorders of the respiratory system**		**08/14**			**04/08**	**01/04**
MDC Respiratory system	14		✗	✓	✓	✗
Respiratory system (as primary diagnosis)	4, 16		✓	✗	✓	✓
chronic disease: respiratory	13, 18		✗	✓	✓	✗
**Obstructive airway diseases**		**05/14**			**02/05**	**01/02**
Asthma/COAD	12		✗	✓	✗	✗
**COPD**		**04/14**			**02/04**	**01/02**
COPD	9		✓	✓	✗	✗
Chronic obstructive pulmonary disease (Charlson comorbidity index)	11		✗	✓	✓	✗
Chronic obstructive pulmonary disease (Charlson comorbidity index)	6		✗	✓	✗	✗
CCM COPD (patients actively enrolled or previously enrolled in COPD CCM program within past 12 months)	2		✓	✗	✓	✓

**Diseases and disorders of the genitourinary system**		**06/14**				**04/06**		**02/04**
Genitourinary system (as primary diagnosis)	4, 16		✓	×	✓		×	
chronic disease: genitourinary	18		×	✓	×		×	
**Kidney and urinary tract disease**		**04/14**				**03/04**		**02/03**
MDC kidney and urinary tract	14		×	✓	✓		✓	
**Kidney disease**		**03/14**				**02/03**		**01/02**
Kidney disease (Charlson comorbidity index)	6		×	✓	✓		×	
History of renal failure	5		✓	✓	✓		✓	
**Acute**		**01/14**				**00/01**		**00/00**
Acute kidney injury	15		✓	n.a.	×		×	
**Chronic**		**01/14**				**00/01**		**00/00**
Chronic kidney disease	15		✓	n.a.	×		×	
**Diseases and disorders of the digestive system**		**05/14**				**02/05**		**01/02**
Main diagnosis classified in Chapter XI ICD 10 - Digestive system disease	6		×	✓	✓		×	
MDC digestive system	14		×	✓	✓		✓	
Gastrointestinal system (as primary diagnosis)	4, 16		✓	×	✓		✓	
chronic disease: gastroenteric	18		×	✓	×		×	
**Peptic ulcer**		**02/14**				**00/02**		**00/00**
Peptic ulcer (Charlson comorbidity index)	11		×	✓	×		×	
Peptic ulcer (Charlson comorbidity index)	6		×	✓	×		×	
**Diseases and Disorders of the Endocrine, Nutritional And Metabolic System**		**10/14**				**06/10**		**03/06**
Endocrine-metabolic system (as primary diagnosis)	4, 16		✓	×	✓		×	
**Obesity/cachexia**		**03/14**				**02/03**		**00/02**
Weight (obese and anorexia)	4		✓	×	✓		×	
Extreme weight (underweight or obese)	15		✓	n.a.	×		×	
**Obesity**		**02/14**				**01/02**		**00/01**
Obesity	14		×	✓	✓		×	
Bariatric patient	4		✓	×	×		×	
**Cachexia**		**01/14**				**01/01**		**00/01**
Cachexia	14		×	✓	✓		×	
**Hyperlipidaemia**		**03/14**				**01/03**		**01/01**
Hyperlipidaemia	12		×	✓	✓		✓	
Hyperlipidaemia	4		✓	×	×		×	
Hyperlipidaemia	15		✓	n.a.	×		×	
**Diabetes mellitus**		**08/14**				**04/08**		**02/04**
Diabetes mellitus (Charlson comorbidity index)	6		×	✓	✓		✓	
Diabetes mellitus (Charlson comorbidity index)	11		×	✓	×		×	
Diabetes	9		✓	✓	×		×	
Diabetes	12		×	✓	✓		×	
CCM Diabetes (patients actively enrolled or previously enrolled in diabetes CCM program within past 12 months)	2		✓	×	✓		✓	
Diabetes	15		✓	n.a.	×		×	
chronic disease: diabetes	18		×	✓	×		×	
History of diabetes	5		✓	✓	✓		×	

**Diseases and disorders of the Musculoskeletal System and Connective Tissue**		**05/14**				**03/05**		**01/03**
MDC musculoskeletal system and connective tissue	14		×	✓	✓		✓	
Connective tissue disease (Charlson comorbidity index)	6		×	✓	×		×	
Musculoskeletal-integumentary system (as primary diagnosis)	4		✓	×	✓		×	
chronic disease: muskuloskeletal	18		×	✓	×		×	
**Arthritis**		**01/14**				**01/01**		**00/01**
Arthritis/ osteoarthritis	12		×	✓	✓		×	
**Osteoporosis**		**01/14**				**00/01**		**00/00**
Osteoporosis	12		×	✓	×		×	
**Diseases and disorders of the hepatobiliary system and pancreas**		**05/14**				**04/05**		**01/04**
MDC7 Hepatobiliary system and pancreas	14		×	✓	✓		×	
**Liver diseases**		**04/14**				**03/04**		**01/03**
Liver diseases (Charlson comorbidity index)	11		×	✓	✓		×	
Liver disease	9		✓	✓	✓		✓	
Liver disease (Charlson comorbidity index)	6		×	✓	✓		×	
Liver diseases	12		×	✓	×		×	
**Neoplasms**		**06/14**				**02/06**		**02/02**
Main diagnosis classified in Chapter II ICD 10 - Neoplasms	6		×	✓	✓		✓	
Neoplasia (Charlson comorbidity index)	6		×	✓	×		×	
Cancer (Charlson comorbidity index)	11		×	✓	✓		✓	
Active cancer	15		✓	n.a.	×		×	
chronic disease: cancer	18		×	✓	×		×	
History of cancer	5		✓	✓	n.a.		×	
Malignancy	12		×	✓	×		×	
**Neoplasms of the Blood and Blood Forming Organs**		**01/14**				**00/01**		**00/00**
Leukemia (Charlson comorbidity index)	6		×	✓	×		×	
Lymphoma (Charlson comorbidity index)	6		×	✓	×		×	
**Metastases**		**01/14**				**00/01**		**00/00**
Metastases (Charlson comorbidity index)	6		×	✓	×		×	
**Pregnancy, cildbirth, and puerperium**		**02/14**				**01/02**		**00/01**
MDC Pregnancy, childbirth, and puerperium	14		×	✓	✓		×	
Pregnancy/ breastfeeding	4		✓	×	×		×	
**HIV**		**02/14**				**00/02**		**00/00**
History of HIV	5		✓	✓	×		×	
**AIDS**		**01/14**				**00/01**		**00/00**
Aids (Charlson comorbidity index)	6		×	✓	×		×	
**Infectious Diseases**		**02/14**				**00/02**		**00/00**
Infection	15		✓	n.a.	×		×	
Infection (UTI/Chest infection)	12		×	✓	×		×	
**Others (named <2x)**		**06/14**				**03/06**		**02/03**
Other (all other diagnoses combined)	16		✓	×	✓		×	
MDC 0: others	14		×	✓	✓		✓	
In-hospital death	6		×	✓	×		×	
Dyspnea (present)	11		×	✓	✓		✓	

Had a rash in the past 2 weeks	5	✓	✓	×	×
Had a seizure in the past 2 weeks	5	✓	✓	×	×

Laboratory value-related risk factor	Citations	Assessed in (number of studies)	Considered for evaluation as risk factor<br>By literature search/expert suggestions	 By statistical method	Tested for inclusion in final scoring tool (e.g. by multivariate analysis due to expert consensus or significance in uni-/bivariate analysis)	Risk factor included in final predicitve scoring tool
**Reduced renal function**		**12/14**			**10/12**	**07/10**
Poor renal function (patients with one of the following test results: eGFR of <30 mL/min/1.73 m² in past 5 days, SCr >200 mmol/L (>2.2 mg/dL) in past 5 days)	2		✓	✗	✓	✓
Deteriorating renal function (rising urea or SCr concentration to >2 times baseline (baseline = mean of all values in past 5 days))	2		✓	✗	✓	✓
**eGFR**		**08/14**			**07/08**	**04/07**
Estimated glomerular filtration rate/10 (ml/min/1.73 m2)	4, 16		✓	✗	✓	✓
Reduced renal function (eGFR > 60)	10		✓	✗	✓	✓
Reduced renal function (60 > eGFR > 30)	10		✓	✗	✓	✓
Reduced renal function (eGFR < 30)	10		✓	✗	✓	✓
Renal failure (GFR <60 ml/min)	9		✓	✓	✓	✓
Renal failure	14		✗	✓	✓	✗
Renal function impairment (eGFR < 30 ml/min/1.73 m²)	8		✓	✓	✓	✓
CKD-EPI (ml/min/1,73 m^2) (severely impaired vs. normal)	1		✓	✓	✓	✗
CKD-EPI (ml/min/1,73 m^2) (moderately impaired vs. normal)	1		✓	✓	✓	✗
eGFR 30-50 ml/min	3, 15		✓	✗	✓	✗
eGFR < 30 ml/min	3, 15		✓	✗	✓	✗
eGFR > 30% change during admission	3, 15		✓	✗	✓	✗
Renal impairment (<60 mLs/min)	12		✗	✓	✗	✗
**Serum creatinine**		**03/14**			**02/03**	**01/02**
Creatinine ≥ 1.4 mg/dl	6		✗	✓	✓	✗
Creatinine ≥ 3.0 mg/dl	11		✗	✓	✗	✗
Creatinine ≥ 150 mmol/L (> 1,7 mg/dL)	5		✓	✓	✓	✓
**Serum urea**		**01/14**			**01/01**	**01/01**
Serum urea ≥ 67 mg/dl	6		✗	✓	✓	✓
**Reduced liver function/ liver failure**		**05/14**			**05/05**	**01/05**
Liver failure	14		✗	✓	✓	✗
Liver disease (ALT/ALP and/or bilirubin ≥ 3 times normal range)	4, 16		✓	✗	✓	✗
Alkaline phosphatase ≥ 85 U/l	6		✗	✓	✓	✗
AST ukat/L	6		✗	✓	✗	✗
ALT ukat/L	6		✗	✓	✗	✗
Gamma GT ≥ 36 UI/l	6		✗	✓	✓	✗
Bilirubin mg/dl	6		✗	✓	✗	✗
Liver failure	12		✓	✗	✓	✗
Liver failure (transaminase levels > twice the upper limit of normal)	8		✓	✓	✓	✓

**Serum albumin**		**05/14**			**02/05**	**00/02**
Albumin < 3.5 g/dl	9		✓	✓	✗	✗
Serum albumin	4, 16		✓	✗	✓	✗
Albumin < 3.1 g/dl	6		✗	✓	✓	✗
Albumin (g/L)	1		✓	✓	✗	✗
Albumin < 25 g/L	15		✓	n.a.	✗	✗
**Serum potassium**		**05/14**			**03/05**	**02/03**
Serum potassium ≥ 4.9 mmol/l	6		✗	✓	✓	✓
Serum potassium	4, 16		✓	✗	✓	✗
Potassium (mmol/L) (low vs. normal)	1		✓	✓	✗	✗
Potassium (mmol/L) (high vs. normal)	1		✓	✓	✗	✗
Potassium (patients with potassium concentration of <3 or >6 mmol/L (<3 or >6 meq/L) in past 5 days)	2		✓	✗	✓	✓
Low potassium (mmol/L)	15		✓	n.a.	✗	✗
High potassium (mmol/L)	15		✓	n.a.	✗	✗
**Serum sodium**		**05/14**			**05/05**	**03/05**
Serum sodium ≥ 141 mmol/l	6		✗	✓	✓	✓
Sodium (≤ 125mml/L)	3, 15		✓	✓	✓	✓
High sodium (mmol/L)	15		✓	n.a.	✗	✗
Serum sodium	4, 16		✓	✗	✓	✗
Sodium (mmol/L) (low vs. normal)	1		✓	✓	✓	✗
Sodium (mmol/L) (high vs. normal)	1		✓	✓	✗	✗
Sodium (patients with sodium concentration of <125 or >155 mmol/L (<125 or >155 meq/L) in past 5 days)	2		✓	✗	✓	✓
**Blood count**		**07/14**			**05/07**	**03/05**
**Hemoglobin**		**06/14**			**03/06**	**01/03**
Hemoglobin < 12 g/dl	6		✗	✓	✓	✗
Anaemia (Hb < 12 g/dl)	9		✓	✓	✗	✗
Anaemia/ haemoglobin	4		✓	✗	✗	✗
Hemoglobin (mmol/L) (low vs. normal and high)	1		✓	✓	✓	✗
Hemoglobin drop > 25% during patient admission	15		✓	n.a.	✗	✗
Hematology (bleeding) (patients with drop in Hb to >25% of baseline (baseline = mean of Hb values over past 5 days) or drop in PCV (packed cell volume) to >25% of baseline (baseline = mean volume over past 5 days)	2		✓	✗	✓	✓
**Platelets**		**04/14**			**02/04**	**01/02**
Platelet count	4, 16		✓	✗	✓	✗
Platelets /ml	6		✗	✓	✗	✗
Platelet count < 50 x 10^9/L	15		✓	n.a.	✗	✗
platelet count of <50 × 10^{9}/L within past 5 days)	2		✓	✗	✓	✓
**Leukocytes**		**06/14**			**03/06**	**03/03**
Infection risk (patients with one of the following test results: WBC count of <3 × 109/L (<3 × 103/mm3) in past 5 days, neutrophil count of <1.5 × 109/L (<1.5 × 103/mm3) in past 5 days)	2		✓	✗	✓	✓
White cell count (10^9/L)	4, 16		✓	✗	✓	✓

High WCC on admission	12		×	✓	✓		✓	
Leukocytes/ ml	6		×	✓	×		×	
Leucocytes (10^9/L)	1		✓	✓	×		×	
White cell count < 3 x 10^9/L	15		✓	n.a.	×		×	
White cell count > 11 x 10^9/L	15		✓	n.a.	×		×	
**Neutrophil count**		**01/14**				**00/01**		**00/00**
Neutrophil count < 1.5 x 10^9/L	15		✓	n.a.	×		×	
**Coagulation**		**05/14**				**02/05**		**02/02**
Coagulation risk (patients with one of the following test results: INR of >3.5 in past 5 days, aPTT of >100 sec in past 5 days)	2		✓	×	✓		✓	
**INR**		**04/14**				**01/04**		**01/01**
INR (>3)	3, 15		✓	✓	✓		✓	
Subtherapeutic INR	15		✓	n.a.	×		×	
INR	6		×	✓	×		×	
Prothrombin time/ INR	4		✓	×	×		×	
INR (ratio)	1		✓	✓	×		×	
**aPTT**		**01/14**				**01/01**		**00/01**
aPTT (>100)	3, 15		✓	✓	✓		×	
Subtherapeutic aPTT	15		✓	n.a.	×		×	
**Diabetes management**		**05/14**				**03/05**		**01/03**
Diabetes management (HbA1c) (patients with one of the following test results: HbA1c of >64 mmol/mol in past 90 days, serum glucose of >11 mmol/L (>198 mg/dL) in last 5 days)	2		✓	×	✓		✓	
Blood glucose/ HbA1c	4		✓	×	×		×	
**Glucose**		**04/14**				**03/04**		**01/03**
Serum glucose (< 11 mmol/l)	3, 15		✓	✓	✓		×	
Random blood glucose levels (maximum level)	15		✓	n.a.	×		×	
Fasting blood glucose levels (minimum level)	15		✓	n.a.	×		×	
Fasting blood glucose levels (maximum level)	15		✓	n.a.	×		×	
Glucose (mmol/L)	1		✓	✓	✓		×	
Glycemic control (patients with glucose concentration of <4 mmol/L (72 mg/dL) in past 5 days and received diabetic medication from Pyxis)	2		✓	×	✓		✓	
Blood sugar ≤ 3 mmol/L	5		✓	✓	×		×	
**HbA1c**		**01/14**				**00/01**		**00/00**
HbA1c (maximum level)	15		✓	n.a.	×		×	
**Positive microbiological tests**		**03/14**				**01/03**		**01/01**
Positive microbiological blood culture	1		✓	✓	×		×	
**Clostridium difficile**		**02/14**				**01/02**		**01/01**
Clostridium difficile (patients with positive Clostridium difficile toxin culture in past 5 days)	2		✓	×	✓		✓	
Clostridium difficile toxin positive culture	15		✓	n.a.	×		×	
**Others (named <2x)**		**05/14**				**03/05**		**02/03**
Bicarbonate ≥ 27 mmol/l	6		×	✓	✓		×	
Urinary flow (24h)	6		×	✓	×		×	

Serum amylase	4	✓	✗	✗	✗
Thyroid function	4	✓	✗	✗	✗
Serum calcium	4	✓	✗	✗	✗
Serum C-reactive protein	4	✓	✗	✗	✗
Number of biochemical tests (≥ 20 vs. < 20)	1	✓	✓	✓	✓
Therapeutic monitoring (patients with one of the following drug concentrations in past 3 days: gentamicin trough of >1.0 mg/L (>1.0 mg/mL), tobramycin trough of >1.0 mg/L (>1.0 mg/mL), amikacin trough of >1.0 mg/L (>1.0 mg/mL), vancomycin trough of >25 mg/L (>25 mg/mL), phenytoin of >80 mmol/L (>20 mg/mL), digoxin of >2.0 nmol/L (>1.5 ng/mL))	2	✓	✗	✓	✓
Myocardial damage (Troponin I) (patients with two troponin values of >300 ng/mL in past 5 days)	2	✓	✗	✓	✓
Warfarin monitoring (patients with warfarin removed from Pyxis during current admission and two consecutive INR values of <1.5 in past 5 days and/or aPTT of <60 sec)	2	✓	✗	✓	✓
Last blood work: ≤ 7 days	5	✓	✓	n.a.	✗
Last blood work: 1-4 weeks	5	✓	✓	n.a.	✗
Last blood work: 1-3 months	5	✓	✓	n.a.	✗
Last blood work: ≥ 3 months	5	✓	✓	n.a.	✗

			Considered for evaluation as risk factor			
**Vital sign-related risk factor**	**Citations**	**Assessed in (number of studies)**	**By literature search/expert suggestions**	**By statistical method**	**Tested for inclusion in final scoring tool (e.g. by multivariate analysis due to expert consensus or significance in uni-/bivariate analysis)**	**Risk factor included in final predicitve scoring tool**
**Consciousness**		**03/14**			**02/03**	**01/02**
Glasgow score ≥ 14	6		✗	✓	✓	✗
Consciousness (clear)	11		✗	✓	✓	✓
Glasgow Coma Scale	12		✗	✓	✗	✗
**Temperature**		**02/14**			**00/02**	**00/00**
Temperature	6		✗	✓	✗	✗
Temperature	4		✓	✗	✗	✗
**Heart rate**		**03/14**			**01/03**	**01/01**
Heart rate	4		✓	✗	✗	✗
Heart rate ≥ 72 bpm	6		✗	✓	✓	✓
Initial heart rate	5		✓	✓	✗	✗
**Blood pressure**		**03/14**			**01/03**	**01/01**
Blood pressure	4		✓	✗	✗	✗
Initial blood pressure	5		✓	✓	✗	✗
**Systolic blood pressure**		**01/14**			**01/01**	**01/01**
Systolic blood pressure ≥ 148 mmHg	6		✗	✓	✓	✓
**Diastolic blood pressure**		**01/14**			**01/01**	**01/01**
Diastolic blood pressure < 79 mmHg	6		✗	✓	✓	✓
**Others (named <2x)**		**03/14**			**00/03**	**00/00**
Respiratory rate	6		✗	✓	✗	✗
Oxygen saturation (%)	1		✓	✓	✗	✗
Patient looks dehydrated	5		✓	✓	✗	✗

			Considered for evaluation as risk factor			
**Patient-related risk factor**	**Citations**	**Assessed in (number of studies)**	**By literature search/expert suggestions**	**By statistical method**	**Tested for inclusion in final scoring tool (e.g. by multivariate analysis due to expert consensus or significance in uni-/bivariate analysis)**	**Risk factor included in final predicitve scoring tool**
**Age**		**14/14**			**10/14**	**06/10**
Age > 58 years	6		✗	✓	✓	✗
(Age/100)^2	7		✗	✓	✓	✓
(Age/100)^3	7		✗	✓	✓	✓
Age	4, 16		✓	✗	✓	✗
Age > 60 years	14		✗	✓	✓	✓
Age ≥ 65 years	11		✗	✓	✓	✗
Age (as part of CCI)	10		✓	✗	✓	✗
Age 18-59 years	10		✓	✗	✓	✗
Age 60-69 years	10		✓	✗	✓	✗
Age 70-79 years	10		✓	✗	✓	✗
Age > 80 years	10		✓	✗	✓	✗
Age ≥ 80 years	9		✓	✓	✗	✗
Age > 70 years	8		✓	✓	✓	✓
Age	1		✓	✓	✓	✓
Age >75 years (non-Maori and non-Pacific Island patients)	2		✓	✗	✓	✓
Age > 65 years (Maori and Pacific Island patients)	2		✓	✗	✓	✓
≥ 65 years old	15		✓	n.a.	✗	✗
≥ 70 years old	15		✓	n.a.	✗	✗
≥ 75 years old	15		✓	n.a.	✗	✗
Age	19		✗	✓	✗	✗
Age > 80 years	5		✓	✓	✓	✓
Age	12		✗	✓	✗	✗
**Sex**		**10/14**			**05/10**	**02/05**
Gender	4		✓	✗	✗	✗
Gender	1		✓	✓	✗	✗
**Female**		**07/14**			**04/07**	**02/04**
Female	7		✗	✓	✓	✗
Female	6		✗	✓	✓	✓
Female	14		✗	✓	✓	✗
Female	8		✓	✓	✓	✓
gender (female)	19		✗	✓	✗	✗
Female	5		✓	✓	✗	✗
Gender (female)	12		✗	✓	✗	✗
**Male**		**03/14**			**03/03**	**00/03**

Male	14		✗	✓	✓		✗	
Male	7		✗	✓	✓		✗	
Male	11		✗	✓	✓		✗	
**Other gender**		**01/14**				**00/01**		**00/00**
Transgender	4		✓	✗	✗		✗	
**Weight**		**03/14**				**01/03**		**00/01**
Weight ≥ 73 kg	6		✗	✓	✓		✗	
Weight/height related factors	4		✓	✗	✗		✗	
Significant weight changes	4		✓	✗	✗		✗	
weight	19		✗	✓	✗		✗	
**BMI**		**03/14**				**01/03**		**00/01**
BMI ≤ 18.5	9		✓	✓	✗		✗	
BMI 18.5-24.9	9		✓	✓	✗		✗	
BMI ≥ 25.0	9		✓	✓	✗		✗	
BMI	16		✓	✗	✓		✗	
BMI	6		✗	✓	✗		✗	
**Ethnicity**		**04/14**				**01/04**		**00/01**
Ethnicity	4		✓	✗	✗		✗	
**Specific**		**03/14**				**01/03**		**00/01**
Asian/Japanese race	11		✗	✓	✓		✗	
Black	6		✗	✓	✗		✗	
Ethnic Origin (White-British)	12		✗	✓	✗		✗	
**Misuse of substances**		**05/14**				**00/05**		**00/00**
**Drugs**		**03/14**				**00/03**		**00/00**
User of i.v. substances	6		✗	✓	✗		✗	
Recreational drugs/ substance misuse	4		✓	✗	✗		✗	
Overdose risk/ previous overdose/ misuse of medication	4		✓	✗	✗		✗	
Use of recreationals drugs	5		✓	✓	n.a.		✗	
**Alcohol**		**03/14**				**00/03**		**00/00**
Alcohol related	4		✓	✗	✗		✗	
Alcohol use/ misuse	4		✓	✗	✗		✗	
Alcohol intake (g)	6		✗	✓	✗		✗	
Alcohol	12		✗	✓	✗		✗	
**Smoking**		**04/14**				**00/04**		**00/00**
Smoking (pack-years)	6		✗	✓	✗		✗	
Smokers	9		✓	✓	✗		✗	
Smoking status/ nicotine use	4		✓	✗	✗		✗	
Smoking	12		✗	✓	✗		✗	
**Falls**		**05/14**				**01/05**		**01/01**
Falls	9		✓	✓	✗		✗	
≥ 1 fall in the last year	8		✓	✓	✓		✓	
Falls risk	4		✓	✗	✗		✗	
Falls or high risk of falls	15		✓	n.a.	✗		✗	
Fall	12		✗	✓	✗		✗	

**Living situation**		**05/14**		**00/05**	**00/00**
Dependent living situation	4	✓	✗	✗	✗
Housing status (e.g. homeless)	4	✓	✗	✗	✗
Member of travelling community	4	✓	✗	✗	✗
Elderly living alone	4	✓	✗	✗	✗
Housebound	4	✓	✗	✗	✗
Living in own home	8	✓	✗	✗	✗
Living situation (dependant living situation, residential aged care facility)	15	✓	n.a.	✗	✗
Where does the patient live: Home alone	5	✓	✓	✗	✗
Where does the patient live: Home with friends or family	5	✓	✓	✗	✗
Where does the patient live: Homeless	5	✓	✓	✗	✗
Where does the patient live: Shelter	5	✓	✓	✗	✗
Where does the patient live: Hotel	5	✓	✓	✗	✗
Where does the patient live: Group Home	5	✓	✓	✗	✗
Where does the patient live: Nursing Home	5	✓	✓	✗	✗
Where does the patient live: Senior's Residence	5	✓	✓	✗	✗
Living alone	12	✗	✓	✗	✗
**Adherence/compliance**		**04/14**		**02/04**	**01/02**
Adherence/compliance	4	✓	✗	✗	✗
Non-compliance with medication	4	✓	✗	✗	✗
Compliance aid	4	✓	✗	✗	✗
Poor medication compliance (patients identified via medication reconciliation process as having comprehension difficulties or as poorly compliant with medications within previous 12 months)	2	✓	✗	✓	✓
Suspected non-adherence	15	✓	n.a.	✗	✗
Compliant with presciption medication	5	✓	✓	✓	✗
**Language**		**03/14**		**01/03**	**01/01**
Non-native speaker	4	✓	✗	✗	✗
Language barrier	4	✓	✗	✗	✗
English difficulty (patients identified via medication reconciliation process as having poor English comprehension or English as a second language within previous 12 months)	2	✓	✗	✓	✓
Non English-speaking background	15	✓	n.a.	✗	✗
**Swallowing problems or nil by mouth**		**02/14**		**00/02**	**00/00**
Swallowing problems	4	✓	✗	✗	✗
Nil by mouth or swallowing difficulties	15	✓	n.a.	✗	✗
Nil by mouth/ enteral tube	4	✓	✗	✗	✗
**Disabilities**		**05/14**		**02/05**	**01/02**
Disability	4	✓	✗	✗	✗
**Mental/intelectual**		**03/14**		**01/03**	**00/01**
Intelectual disability/learning difficulty	4	✓	✗	✗	✗
Capacity as defined by Mental Capacity Act	4	✓	✗	✗	✗
Cognitive function/mental capacity/mental health status/confusion/delirium	4	✓	✗	✗	✗
Confusion	5	✓	✓	✓	✗

Confusion	12	✗	✓	✗	✗
Cognition (AMTS)	12	✗	✓	✗	✗
**ADL impairment**		**04/14**		**01/04**	**01/01**
ADL disability	9	✓	✓	✗	✗
Needs assistance for ≥ 1 ADL (activity of daily living)	8	✓	✓	✓	✓
ADL score/functional level	4	✓	✗	✗	✗
Barthel Activity of Daily Living	12	✗	✓	✗	✗
**Other disabilities**		**02/14**		**01/02**	**00/01**
Impaired manual skills	4	✓	✗	✗	✗
Visual impairment	4	✓	✗	✗	✗
Physical/ sensory impairment	4	✓	✗	✗	✗
Ability to sign consent form	4	✓	✗	✗	✗
Requires assistance in taking medications	5	✓	✓	✓	✗
**Medical procedures/equipment**		**03/14**		**01/03**	**00/01**
Home oxygen therapy	11	✗	✓	✓	✗
Hemodialysis	11	✗	✓	✗	✗
Mechanical ventilation	6	✗	✓	✗	✗
Venous access patinet/type of cannula	4	✓	✗	✗	✗
**Others (named <2x)**		**03/14**			
Frailty score	4	✓	✗	✗	✗
Marital status	4	✓	✗	✗	✗
Socioeconomic status (based on the English indices of deprivation 2015 (Index of Multiple Deprivation Rank))	16	✓	✗	✓	✗
Social/ cultural issues	4	✓	✗	✗	✗
Patient health beliefs/ behaviours	4	✓	✗	✗	✗
Carer status	4	✓	✗	✗	✗
Nutritional status	4	✓	✗	✗	✗
Decanting of medicines occurring	4	✓	✗	✗	✗
Poor health literacy	4	✓	✗	✗	✗
Patient education level/ literacy	4	✓	✗	✗	✗
Patient/carer level of knowledge/ patient baseline understanding of disease state/ medication	4	✓	✗	✗	✗
Pain score	4	✓	✗	✗	✗
Height ≥ 159 cm	6	✗	✓	✓	✗
Organ transplant	15	✓	n.a.	✗	✗
Poor historian	15	✓	n.a.	✗	✗
Patient described as frail by any member of the healthcare team	15	✓	n.a.	✗	✗

			Considered for evaluation as risk factor			
**Medication process/ setting-related risk factor**	**Citations**	**Assessed in (number of studies)**	**By literature search/expert suggestions**	**By statistical method**	**Tested for inclusion in final scoring tool (e.g. by multivariate analysis due to expert consensus or significance in uni-/bivariate analysis)**	**Risk factor included in final predicitve scoring tool**
**Type of admission**		**07/14**			**06/07**	**03/06**
**Surgical admission**		**03/14**			**03/03**	**01/03**
Surgical admission	7		×	✓	✓	✓
Surgical admission	14		×	✓	✓	×
Surgical admission	11		×	✓	✓	×
**Medical admission**		**03/14**			**03/03**	**00/03**
Medical admission	11		×	✓	✓	×
Medical admission	14		×	✓	✓	×
Medical admission	7		×	✓	✓	×
**Intensive care/critical care admission**		**02/14**			**01/02**	**00/01**
Critical care admission	4		✓	×	×	×
ICU admission	11		×	✓	✓	×
**Scheduled/unplanned admission**		**06/14**			**04/06**	**02/04**
Admission (emergency vs. elective)	1		✓	✓	✓	×
Elective versus unplanned admission	4		✓	×	×	×
**Scheduled admission**		**02/14**			**01/02**	**00/01**
Scheduled admission	14		×	✓	×	×
Scheduled admission	11		×	✓	✓	×
**Emergency admission**		**04/14**			**03/04**	**02/03**
Emergency admission	11		×	✓	✓	×
Admission from emergency room	7		×	✓	✓	✓
Urgent admission	14		×	✓	×	×
at least urgent admission by ambulance arrival (CTAS ≤ 3)	5		✓	✓	✓	✓
**Drug-related admission**		**02/14**			**00/02**	**00/00**
Medicines-related admission	4		✓	×	×	×
Drug related admission	15		✓	n.a.	×	×
**Other admission**		**05/14**			**03/05**	**01/03**
Social-related admission	4		✓	×	×	×
Other admission	11		×	✓	✓	×
New admission (not readmitted)	3		✓	✓	✓	✓
Department of admission: General Surgery vs. Urology	1		✓	✓	✓	×
Department of admission: Orthopedic S vs. Urology	1		✓	✓	✓	×
Admission from an outside institution	7		×	✓	✓	✓
**Previous hospital admission/readmission**		**07/14**			**03/07**	**02/03**
Readmission due to prior admission	14		×	✓	✓	×
Readmission to hospital within 30 days	4		✓	×	×	×

Readmission (within 12 months)	3, 15		✓	✓	✓		×
Readmission in previous 7 days (All currently admitted patients with prior discharge in prev	2		✓	×	✓		✓
Readmission in previous 30 days (All currently admitted patients with prior discharge in pre	2		✓	×	✓		✓
Hospital admission within previous 30 days	7		×	✓	✓		✓
Last hospitalization: ≤ 7 days	5		✓	✓	✓		✓
Last hospitalization: 1-4 weeks	5		✓	✓	✓		✓
Last hospitalization: 1-3 months	5		✓	✓	×		×
Last hospitalization: > 3 months	5		✓	✓	×		×
Previous hospital admission	12		×	✓	×		×
**Type of hospital department/ specialty**		**02/14**				**01/02**	**01/01**
Type of hospital department/ specialty	4		✓	×	×		×
**High risk specialty**		**01/14**				**01/01**	**01/01**
High-risk specialty (patients under care of hematology or renal service (excludes patients ad	2		✓	×	✓		✓
**Time of admission/ stay/ prescription**		**02/14**				**01/02**	**00/01**
Day-time admission (9 AM to 8 PM)	7		×	✓	✓		×
Night-time admission (8 PM to 9 AM)	7		×	✓	✓		✓
Weekday admission	7		×	✓	✓		×
Weekend (or holiday) admission	7		×	✓	✓		×
Time of day prescribed	4		✓	×	×		×
Month of stay	4		✓	×	×		×
The days admitted/ time of year	4		✓	×	×		×
**Number of previous hospitalizations**		**02/14**				**01/02**	**00/01**
Number of previous hospitalizations	6		×	✓	×		×
Number of hospital admissions in the previous 6 months	4, 16		✓	×	✓		×
**Length of stay**		**03/14**				**02/03**	**02/02**
Length of stay ≥12 days	12		×	✓	✓		✓
LOS Length of stay (days)	3		✓	✓	✓		✓
Length of stay	4		✓	×	×		×
**Medical changes before admission**		**03/14**				**02/03**	**02/02**
Cessation of medicines used before admission	4		✓	×	×		×
Prescription of new medicines during/ before admission	4		✓	×	×		×
Treatment initiated before admission	7		×	✓	✓		✓
Medication changes within 28 days	5		✓	✓	✓		✓
**Transfer across units/wards**		**04/14**				**02/04**	**01/02**
Transfer from other unit within 72 hours	7		×	✓	✓		×
Number of patient transfers across wards	4		✓	×	×		×
High-risk transfer (patients transferred from ICU, HDU, or CCU to another ward location wit	2		✓	×	✓		✓
Transfer from intensive care unit or high dependency unit	15		✓	n.a.	×		×
**Outpatient visits**		**02/14**				**01/02**	**01/01**
Number of outpatient appointments within 6 months	4		✓	×	×		×
Multi outpatient visits (patients with >2 outpatient visits to different specialties in previous	2		✓	×	✓		✓
**General practitioner**		**02/14**				**01/02**	**01/01**
Regular general practitioner	5		✓	✓	n.a.		×
No registered General Practitioner (during most recent prior admission within previous 12 ı	2		✓	×	✓		✓

Others (named <2x)	05/14			04/05	02/04
Stage of patient stay (admission/ during stay/ discharge)	4	✓	✗	✗	✗
Identifying if patient is on a risk register with general practitioner	4	✓	✗	✗	✗
Frequency of GP contact	4	✓	✗	✗	✗
Staffing levels on ward/ hospital	4	✓	✗	✗	✗
Communication problems across interfaces	4	✓	✗	✗	✗
End of life care	4	✓	✗	✗	✗
Length of time on medicine/newly prescribed	4	✓	✗	✗	✗
Self-care for medicines/ whether patient/ family/ carer is responsible for medicines	4	✓	✗	✗	✗
Homecare provided medicines	4	✓	✗	✗	✗
Resident physician in charge (≤ 3 years of training after obtaining a license)	11	✗	✓	✓	✓
Scheduled operation	11	✗	✓	✓	✓
Number of prescribing physicians: at most one	5	✓	✓	✗	✗
Number of prescribing physicians: two or more	5	✓	✓	✗	✗
Medications blister packed	5	✓	✓	✓	✗
Frequent presenter (patients with >4 Emergency care presentations/inpatient admissions i	2	✓	✗	✓	✓
Best possible medication history available	7	✗	✓	✓	✓

Legend

Risk factor did not increase the risk of a DRP (e.g., counted as 0 points in the final scoring tool) but was included in the final scoring tool.

Risk factor did decrease the risk of a DRP (e.g., counted as -1 point in the final scoring tool) but was included in the final scoring tool.

These risk factors were counted only as "investigated by the authors" even though they were included in a final scoring tool because we focused exclusively on risk factors that increase the risk of a DRP in this review.

Citations

1. (in main text cited as reference 29) Bos, J. M.; Kalkman, G. A.; Groenewoud, H.; Van Den Bemt, P. M. L. A.; De Smet, P. A. G. M.; Nagtegaal, J. E.; Wieringa, A.; Van Der Wilt, G. J.; Kramers, C. Prediction of clinically relevant adverse drug events in surgical patients. *PLOS ONE* **2018,** *13* (8), e0201645. doi:10.1371/journal.pone.0201645.

2. (in main text cited as reference 34) Falconer, N.; Nand, S.; Liow, D.; Jackson, A.; Seddon, M. Development of an electronic patient prioritization tool for clinical pharmacist interventions. *American Journal of Health-System Pharmacy* **2014,** *71* (4), 311-320. doi:10.2146/ajhp130247.

3. (in main text cited as reference 24) Falconer, N.; Barras, M.; Abdel-Hafez, A.; Radburn, S.; Cottrell, N. Development and validation of the Adverse Inpatient Medication Event model (AIME). *British Journal of Clinical Pharmacology* **2020**. doi:10.1111/bcp.14560.

4. (in main text cited as reference 20) Geeson, C.; Wei, L.; Franklin, B. D. Development and performance evaluation of the Medicines Optimisation Assessment Tool (MOAT): a prognostic model to target hospital pharmacists' input to prevent medication-related problems. *BMJ Quality & Safety* **2019,** *28* (8), 645-656. doi:10.1136/bmjqs-2018-008335.

5. (in main text cited as reference 31) Hohl, C. M.; Yu, E.; Hunte, G. S.; Brubacher, J. R.; Hosseini, F.; Argent, C. P.; Chan, W. W. Y.; Wiens, M. O.; Sheps, S. B.; Singer, J. Clinical Decision Rules to Improve the Detection of Adverse Drug Events in Emergency Department Patients. *Academic Emergency Medicine* **2012,** *19* (6), 640-649. doi:10.1111/j.1553-2712.2012.01379.x.

6. (in main text cited as reference 17) Lima, S. I. V. C.; Martins, R. R.; Saldanha, V.; Silbiger, V. N.; Dos Santos, I. C. C.; Araújo, I. B. D.; Oliveira, A. G. Development and validation of a clinical instrument to predict risk of an adverse drug reactions in hospitalized patients. *PLOS ONE* **2020,** *15* (12), e0243714. doi:10.1371/journal.pone.0243714.

7. (in main text cited as reference 21) Nguyen, T.-L.; Leguelinel-Blache, G.; Kinowski, J.-M.; Roux-Marson, C.; Rougier, M.; Spence, J.; Le Manach, Y.; Landais, P. Improving medication safety: Development and impact of a multivariate model-based strategy to target high-risk patients. *PLOS ONE* **2017,** *12* (2), e0171995. doi:10.1371/journal.pone.0171995.

8. (in main text cited as reference 18) O'Mahony, D.; O'Connor, M. N.; Eustace, J.; Byrne, S.; Petrovic, M.; Gallagher, P. The adverse drug reaction risk in older persons (ADRROP) prediction scale: derivation and prospective validation of an ADR risk assessment tool in older multi-morbid patients. *European Geriatric Medicine* **2018,** *9* (2), 191-199. doi:10.1007/s41999-018-0030-x.

9. (in main text cited as reference 22) Onder, G.; Petrovic, M.; Tangiisuran, B.; Meinardi, M. C.; Markito-Notenboom, W. P.; Somers, A.; Rajkumar, C.; Bernabei, R.; Van Der Cammen, T. J. M. Development and Validation of a Score to Assess Risk of Adverse Drug Reactions Among In-Hospital Patients 65 Years or Older. *Archives of Internal Medicine* **2010,** *170* (13). doi:10.1001/archinternmed.2010.153.

10. (in main text cited as reference 26) Saedder, E. A.; Lisby, M.; Nielsen, L. P.; Rungby, J.; Andersen, L. V.; Bonnerup, D. K.; Brock, B. Detection of Patients at High Risk of Medication Errors: Development and Validation of an Algorithm. *Basic & Clinical Pharmacology & Toxicology* **2016,** *118* (2), 143-149. doi:10.1111/bcpt.12473.

11. (in main text cited as reference 27) Sakuma, M.; Bates, D. W.; Morimoto, T. Clinical prediction rule to identify high-risk inpatients for adverse drug events: the JADE Study. *Pharmacoepidemiology and Drug Safety* **2012,** *21* (11), 1221-1226. doi:10.1002/pds.3331.

12. (in main text cited as reference 19) Tangiisuran, B.; Scutt, G.; Stevenson, J.; Wright, J.; Onder, G.; Petrovic, M.; Van Der Cammen, T. J.; Rajkumar, C.; Davies, G. Development and Validation of a Risk Model for Predicting Adverse Drug Reactions in Older People during Hospital Stay: Brighton Adverse Drug Reactions Risk (BADRI) Model. *PLoS ONE* **2014,** *9* (10), e111254. doi:10.1371/journal.pone.0111254.

13. (in main text cited as reference 30) Trivalle, C.; Burlaud, A.; Ducimetière, P. Risk factors for adverse drug events in hospitalized elderly patients: A geriatric score. *European Geriatric Medicine* **2011,** *2* (5), 284-289. doi:10.1016/j.eurger.2011.07.002.

14. (in main text cited as reference 28) Urbina, O.; Ferrández, O.; Grau, S.; Luque, S.; Mojal, S.; Marin-Casino, M.; Mateu-de-Antonio, J.; Carmona, A.; Conde-Estévez, D.; Espona, M.; González, E.; Riu, M.; Salas, E. Design of a score to identify hospitalized patients at risk of drug-related problems. *Pharmacoepidemiology and Drug Safety* **2014,** *23* (9), 923-932. doi:10.1002/pds.3634.

15. (in main text cited as reference 23) Falconer, N.; Barras, M.; Cottrell, N. How hospital pharmacists prioritise patients at high-risk for medication harm. *Research in Social and Administrative Pharmacy* **2019,** *15* (10), 1266-1273. doi:10.1016/j.sapharm.2018.11.003.

16. (in main text cited as reference 36) Geeson, C.; Wei, L.; Franklin, B. D. Medicines Optimisation Assessment Tool (MOAT): a prognostic model to target hospital pharmacists' input to improve patient outcomes. Protocol for an observational study. *BMJ Open* **2017,** *7* (6), e017509. doi:10.1136/bmjopen-2017-017509.

17. Nguyen, T.-L. (University of Copenhagen, Copenhagen, Denmark). Personal Communication, 2022.

18. (in main text cited as reference 43) Trivalle, C.; Cartier, T.; Verny, C.; Mathieu, A. M.; Davrinche, P.; Agostini, H.; Becquemont, L.; Demolis, P. Identifying and preventing adverse drug events in elderly hospitalised patients: A randomised trial of a program to reduce adverse drug effects. *The journal of nutrition, health & aging* **2010,** *14* (1), 57-61. doi:10.1007/s12603-010-0010-4.

19. Trivalle, C. (Hôpital Paul-Brousse AP-HP, Villejuif, Val-de-Marne, France). Personal Communication, 2022.
